# Impact of market diversification and prices on mango exports in emerging economies in the period 2004-2024

**DOI:** 10.12688/f1000research.177965.1

**Published:** 2026-03-05

**Authors:** Ricardo Fernando Cosio Borda, Janel Janelvia Ancajima Velásquez, Percy Hugo Quispe-Farfán, Natalie Margarita Gamarra-Vargas, Esther Rosa Saenz Arenas, Omar Alonso Patiño Castro, Berenice Cajavilca-Gonzáles, Jorge Luis Cardich Pulgar

**Affiliations:** 1 Facultad de Administración, Finanzas y Ciencias Económicas, Universidad EAN, Bogotá, Colombia; 2 Universidad César Vallejo, Piura, Peru; 3 Universidad Peruana de Ciencias Aplicadas, Lima, Peru; 4 Universidad Tecnológica del Perú, Lima, Peru; 5 Universidad San Ignacio de Loyola, Lima, Peru

**Keywords:** Agroindustry, exports, diversification, market, price.

## Abstract

**Background:**

This study analyzed the performance of mango exports in Peru and Colombia during the period 2004-2024, addressing the economic vulnerability generated by concentration in a few destinations and the volatility of international prices. The research suggested that dependence on limited markets and price variations represented critical challenges to the stability of producers in these emerging economies. Previous research on products such as ginger, blueberries, and quinoa in Peru, as well as coffee and avocados in Colombia, was considered. This research highlighted the importance of internal factors and the opening of new markets to improve competitiveness. The main problem was to determine how market diversification and prices impacted mango exports in both countries. The overall objective was to assess this impact to better understand the competitiveness of the sector.

**Method:**

The methodology adopted was applied, with a quantitative approach, non-experimental design, correlational level, and longitudinal scope. Annual data on the number of markets, FOB price, volume, and FOB value were extracted from the Trade Map database for the period 2004-2024. SPSS software was used for statistical processing and hypothesis validation.

**Results:**

The main results indicated that, in the case of Peru, diversification and price together explained 90.7% of the variation in export value. In Colombia, diversification explained 89.9% of the fluctuations in FOB value, while the influence of price was not statistically significant.

**Conclusions:**

The main conclusion established that market diversification was the key factor in both countries, although price only had a significant impact on Peru's export performance.

## 1. Introduction

International trade in agricultural products has undergone a very changing dynamic on a global scale with significant transformations, especially in the last two decades. During 2023, exports of these products were USD1,465.88 million, influenced by the increase in global demand, as well as the phenomenon of globalization ( World Trade Organization, 2023a). This scenario generated challenges and opportunities for producing countries, which, in search of sustainable growth, found themselves in the constant research of marketing strategies ( Brunetto Beretervide et al., 2024). Along these lines, variations in international prices and the need to amplify the geographical destinations of exports became crucial factors in mitigating risks and maintaining economic stability in trade in agricultural products ( World Trade Organization, 2023b).

Particularly, in the mango market, the impact of the aforementioned trends was significantly reflected. In fact, global mango exports in 2023 totaled USD4,252.3 million ( MIDAGRI, 2025), with the growing global demand for these fruits being the main factor that explained these results ( FAO, 2023).

However; Despite the developing dynamics of these exports, scenarios such as the intensification of competition from producing countries were presented, which resulted in a variation in prices and market share. Similarly, while regional and multilateral trade agreements facilitated penetration of new markets, there were logistical and technical obstructions that limited the successful diversification of agricultural products ( FAO, 2023). This scenario implied that, despite the fact that the volume exported increased, the concentration in a few markets and the variation in prices continued to be challenges that impacted the sustainability and profitability of the sector.

In Latin American countries such as Peru and Colombia, mangoes were presented as a product with many opportunities in the international market. In Peru, it was not only thanks to the quality and flavor they presented, but also to the country's ability to offer a large volume at competitive prices, thus satisfying the needs of the market that was inclined towards an improvement in food and whose national market failed to meet its demand ( MIDAGRI, 2022). The Ministry of Agrarian Development and Irrigation highlighted the importance of implementing initiatives that improve the competitiveness of the fruit, in order to take advantage of the opportunities offered by the demand and satisfy the volume and quality of consumers.

On the other hand, Colombian mango exports also reflected a dynamic of expansion and growth, seeking greater penetration in the world market. In 2024, the FOB value of fresh mango exports registered a growth of 13% (January-June) compared to 2023, while its net weight registered a positive variation of 39.6% in the same periods compared ( National Association of Foreign Trade, 2024).

Colombia sought the expansion of new mango destinations through strategies for compliance with phytosanitary and quality rules. This is how, in 2022, the first container of mango was exported to the United States, in a joint effort to obtain authorization from the North American country for the entry of Colombian fruit ( Ministry of Commerce, Industry and Tourism, 2022). It was noted that the strategies articulated between the public and private sectors will continue to improve Colombia's opportunities and its export offer.

The level of participation of mango exports based on the numbers of destination markets, as well as their volume, price and value were highlighted as key aspects to understand the behavior of the competitiveness of this fruit. However, the influence they may have was not sufficiently studied, which limited the understanding of how these variables behaved to impact the performance of the sector.

In this regard, the research was harmonized with Sustainable Development Goal (SDG) 12, which promotes responsible production and consumption. This is relevant because, by focusing on the analysis of diversification and its relationship with prices and exports, the findings were an informed basis capable of being used for the formulation of strategies that promote greater competitiveness and thus a more responsible use of resources. Thus, it sought to contribute to a more sustainable economic development that benefits local producers and the environment.

That is why the general problem was raised: How does market diversification and prices impact mango exports from Peru and Colombia in the period 2004-2024?

For the specific problems, the following questions were raised: 1. What is the relationship between market diversification and mango prices in Peru and Colombia in the period 2004 - 2024? 2. How do market diversification and prices impact Peru's mango exports in the period 2004 - 2024? 3. How do market diversification and prices impact Colombia's mango exports in the period 2004-2024?

The present research was theoretically justified in frameworks such as those of Ansoff, who proposed the theory of market diversification. In the same way, Krugman's theory refers to prices in international trade, as well as Porter's theory based on exports.

The scientific method used in this research was quantitative, with a non-experimental design and a correlational approach, which allowed analyzing how the variables of diversification of markets, prices and exports were related to each other. This approach was appropriate because they worked with historical data from Peruvian mangoes, using statistical tools to validate the hypotheses.

In addition, the results of this research provided a clearer understanding of how market dynamics influenced mango exports. By providing data and analysis on the influence of market and price diversification, it was hoped that the findings will serve as a reference for the formulation of new strategies to boost the competitiveness of the sector. In this way, it will contribute to the economic stability of producers, improving their ability to face market fluctuations and favoring sustainable growth in the country.

As a general objective, it was proposed: To evaluate the impact of market diversification and prices on mango exports from Peru and Colombia in the period 2004 – 2024.

While, as specific objectives, the following were proposed: 1. To identify the relationship between market diversification and mango prices in Peru and Colombia in the period 2004 - 2024. 2. To evaluate the impact of market diversification and prices on Peru's mango exports in the period 2004 - 2024. 3. To evaluate the impact of market diversification and prices on mango exports from Peru and Colombia in the period 2004-2024.

It was essential to learn about different previous research on the study variables applied to the Peruvian context. It was key to highlight the research by Arana et al. (2021) in which the development of ginger exports in the Peruvian scenario, mainly destined for the United States, was contextualized, highlighting how various internal factors were considered in the performance of exports of this product. In this line, the authors set as a general objective to determine the relationship between these internal factors that had such an impact on ginger exports. It was based on Balanko's internal factors model, taking into account price, volume, value and investment in technology and innovation, all corresponding to the period 2006-2020. In the same way, the methodological approach that was presented was quantitative, with a correlational level. The results were a direct relationship between the quantity exported and the price of sale to the foreign market, as well as the relativity between volume and quantity and the relationship between quantity exported and investment in technology and innovation. Consequently, it was determined as a conclusion that it was key to take into account the aforementioned internal factors, in order to ensure optimal results in the exports of ginger to the U.S. market.

Following this line, Montes et al. (2024) took into account the problem of the barriers faced by the sustainability of the international sale of Peruvian blueberries, mainly caused by dependence on the few destination markets, as well as competitiveness strategies for efficient production. Therefore, they aimed to analyze the blueberry trade in the international context, based on the factors of competitiveness and diversification. International trade theory was used, as well as comparative advantage. Regarding the methodology, a quantitative approach and a descriptive design were presented. The results were an excellent growth in the export value of blueberries, as well as a high competitiveness of this fruit for export. Despite this, the United States was by far the main destination market for this product, with growth in the United Kingdom and the Netherlands, thus demonstrating a high concentration, which developed the risk of depending on these markets, specifically the North American country. It was concluded that, although Peru developed with increasing competitiveness in the Peruvian blueberry sector, there was a risk of strong dependence on markets and had the challenge of diversifying its destinations.

Finally, the research by Huacani et al. (2024) studied the problem of the instability of export prices of quinoa produced in Peru, which triggered problems related to demand. In view of this, the objective was to identify the correlation between the export of quinoa and the price set at the port of this product. Theoretically, it was based on the theory of international trade, specifying exports. A methodology with a quantitative and correlational approach was used, using Pearson's correlation. Significant values were obtained as results in the 3 analyses, which shows that there is a correlation between both variables. In this way, it was concluded that the promotion and strengthening of the international marketing of quinoa is of utmost importance, since the international presence of a product originating in Peru is promoted.

With regard to the Colombian market, research such as that of García et al. (2025) was highlighted. with the study that examined the vulnerability faced by coffee-exporting countries in South America, including Colombia, due to their high dependence on a limited number of markets. This situation increasingly exposes them to risks such as climate change and geopolitical instability. The main objective was to describe, through a quantitative analysis, the level of market concentration and international competitiveness of the main South American producers. Thus, it sought to offer solid evidence on the conditions that favor sustainable trade diversification. To achieve this, a comparative quantitative methodology was applied that used the Herfindahl-Hirschman Index (HHI) and the Revealed Comparative Advantage Index (RCA) to analyze the export of unroasted or decaffeinated coffee (item 090111) in the period 2015–2024. The results for Colombia were encouraging. The total value of exports of unroasted and decaffeinated coffee grew significantly, from USD 42.41 million in 2015 to USD 104.74 million in 2024, representing an increase of close to 150%. This growth was accompanied by a progressive diversification of export destinations, evidenced by the reduction of HHI, which fell from 6,765 in 2015 to 4,789 in 2024, showing a 29% decrease in market concentration. In terms of competitiveness, Colombia managed to strengthen its position in regional markets such as Chile and Ecuador. However, in the important U.S. market, its position was more variable and had a net drop. The main conclusion of the study highlighted that effective coordination between trade, infrastructure, and science and technology policies is essential for South American coffee-producing countries to improve their comparative advantage. This is key to ensuring long-term sustainability in the face of both structural and situational challenges. In this sense, Colombia stood out for having one of the most resilient export structures in the region, thanks to a balance between diversification and competitiveness.

Also relevant was the research by Torres and Trochez (2023) that focused on the avocado market, taking the problem of the effects produced by the constant variation in the price of this product according to its varieties and destination market. Its main objective was to carry out a review of international marketing, as well as the prices of this product, related to the FTAs that the country had. They were based on the theory of international trade and prices, taking a mixed linear model in their methodology. The main results showed that the Hass type of avocado suffered an increase in its price, while the other two types studied (papelillo and común) showed a downward trend in it. It was concluded that the Hass avocado has been one of the beneficiaries of the opening of Colombian agricultural products in international markets, increasing the amount exported of this product, as well as the establishment of relations with other nations that will allow taking advantage of the demand that was in development.

Finally, the study by Cubillos et al. (2021) examined how agricultural trade between Colombia and the European Union (EU) has evolved over the period 2008-2019, with a particular focus on three key products: bananas, coffee, and palm oil. Through the use of Pearson's correlation coefficient, the degree of influence that each of these items had on the total value of the country's agricultural exports was evaluated. The results showed that palm oil played a particularly relevant role, showing a positive and significant correlation with the overall value of agricultural exports. This behavior reflected the ability of the product to take advantage of the opportunities generated by trade liberalization and the entry into force of the Free Trade Agreement (FTA) with the EU. Since the implementation of the agreement in 2013, palm oil registered the most sustained growth among the three products, increasing its export value by 252% between 2012 and 2018. In addition, its share of agricultural exports to the EU increased from 3% in 2011 to 12.2% in 2019. The statistical analysis yielded a Pearson coefficient of 0.815, the highest in the set, which evidenced a strong positive relationship between palm oil exports and the overall performance of the Colombian agricultural sector.

To analyze the diversification of markets, prices and exports of Peruvian and Colombian mangoes, several economic theories and international trade models were used to help understand the phenomena that impact this sector.

One of the key theories to understand market diversification was proposed by Igor Ansoff. The theorist outlines a product-market matrix, where the market expansion strategy was presented. In it, Ansoff explained that one way to achieve the economic development of companies is to take a product that already exists or is already produced by an organization to another market and is based on the need or strategy of the company to distribute its goods in a demographic or geographic segment different from the one it already sells. This strategy includes taking moderate risks ( Ferreira & Marques, 2024). In this theoretical framework, the basis taken by companies that want to maintain their value proposition through their products, but crossing new borders and diversifying their destinations, was reflected.

Complementing this theory, Johanson and Wiedersheim presented the famous Uppsala model, where, already having in mind the idea of market expansion as a strategy to be developed, they moved on to the most efficient way to apply diversification. Theorists explained that firms and nations expand gradually, trying first to penetrate markets where demographic, cultural, and economic characteristics are similar to those of the country of origin, moving closer and closer to more distant nations ( Johanson and Wiedersheim, 1975). In this way, the Uppsala model aimed to help companies not only diversify their portfolio of markets, but also to do so by mitigating the risks that may be involved in this process, collecting experience and knowledge to progressively have a better distributed presence in the world.

Finally, Hitt et al. (1997) covered the diversification of markets in a more developed scenario, in which producers, having already diversified into goods, decide to diversify into markets. When analyzing expanding into a considerable number of markets, theorists considered both positive and negative aspects of this strategy.

On the positive side, new information and multiple strategies are collected for the efficiency of product marketing, in addition to the reduction of the unit cost of production due to economies of scale, as well as the reduction of risks due to diversification, mitigating the impact that a crisis would have on one of the target markets ( Hitt et al., 1997).

On the other hand, the challenges of this strategy imply higher costs in terms of logistics or operations management in all destination countries, as well as the difficulty for the information collected in one country to be coupled with that of another ( Hitt et al., 1997). For this reason, the theorists suggested that it is key to identify the points of competitiveness that allow the company to impact its target markets and take advantage of these advantages, in order to ensure success in market diversification.

In another line, the Heckscher-Ohlin model, also known as H-O, was key to understanding the price variable focused on international trade. In this model, the authors explained that, assuming that we are in a scenario of perfect competition, the price of goods, as well as the cost of production, will be conditioned by the endowment and intensity of factors; that is, whether the nation has more capital or labor, and whether they have it at a competent price level. A country that has abundance and an advantage in the cost of capital will specialize in producing and marketing goods or products that require the advantages of this factor, having as a market countries in which manufacturing such products implies a higher cost. On the other hand, countries that have more work (cheaper labour in countries with large populations, for example) will focus on the production of goods that require more of the labour factor and offer more attractive prices to countries where the production of this type of goods is much more expensive. In this way, both countries exchange products in which they are specialized at a competitive price ( Páez et al., 2021).

Subsequently, Krugman complemented the vision of the H-O model, in a modern approach, analyzing the scenario where consumers demanded variety of a product as it penetrated the market, when several countries marketed the same good, but with differentiation, at a lower price. In his theory, Krugman associated international sales prices with two factors: economies of scale and monopolistic competition. Economies of scale, as Krugman suggests, determine that a country that produces a specific good en masse will be able to have cheaper unit costs and, therefore, offer a much cheaper final price ( Posada and Vélez, 2008).

On the other hand, the theorist affirmed that nations have the power to produce a good, but giving it an added value, which is differentiation. When producing and marketing a specific type of product, countries can determine a price since they have "power" over their variety, as long as these prices are not very high taking into account the possible entry of competition, which would be a turning point in the advantage that monopolistic power gives them ( Posada and Vélez, 2008).

Considering that Krugman also mentioned transportation costs as an important factor in production and prices, a vision that outlines logistics costs and how they influence product prices had to be taken into account. This is how Baena and Cano presented a methodology for determining the final price of an export in a contract, based on the famous Incoterms, which, through their terms, give an indicator of the responsibilities, risks and costs assumed by both sellers and buyers, depending on the various scenarios that are taken in the process of transporting the goods. In this model, the authors add up the production costs, profit margin and transportation costs resulting in a final price for each incoterm used (EXW, FOB, CIF, etc.). In this way, it was possible to understand how trade is evolving and the factors to be taken into account for the international price of goods, taking into account the advantages and interests of producers ( Baena and Cano, 2022).

On the other hand, to understand the export variable, the Theory of International Trade was used, which has different approaches.

One of the best-known theories was the theory of absolute advantage, proposed by David Ricardo. The author explained that, although a country can be a good producer of many goods or all of them, it will focus strategically on producing and selling internationally the good that generates the lowest opportunity cost; that is, the good in which it is relatively better ( López, 2012). In this way, countries lose the opportunity to manufacture other products, but they obtain a greater good with those they specialize in and the profits they generate can be useful to acquire other products they need from other countries. This theory was fundamental for the understanding of the dynamics of the exchange of goods between nations and the purpose of this process.

Subsequently, this proposal was reinforced with the H-O model through the factorial ratio, in which, in agreement with Ricardo's proposal, it was explained that countries will follow the trend of exchanging goods in those with greater production specialty, due to comparative advantage granted by nature itself (Peru with certain agricultural products, for example). In this way, the circulation of goods between countries was also explained, giving flow to international trade in exports and imports ( Páez et al., 2021).

Finally, in a more novel field, Michael Porter introduced the theory of competitive advantage. In it, Porter argued that a country's advantage to export is not only about factor endowments or what each country is good at, but about a series of strategies that improve quality, processes and infrastructures that allow its products to have an added value in the world view ( Páez et al., 2021).

As a general hypothesis, it was proposed: There is a significant impact of market diversification and prices on mango exports from Peru and Colombia in the period 2004 – 2024. H0: There is no significant impact of market diversification and prices on mango exports from Peru and Colombia in the period 2004 – 2024.

While, as specific hypotheses, the following were proposed: 1. There is a significant relationship between market diversification and mango prices in Peru and Colombia in the period 2004 - 2024. H0 There is no significant relationship between market diversification and mango prices in Peru and Colombia in the period 2004 - 2024 2. There is a significant impact of market diversification and prices on Peru's mango exports in the period 2004 - 2024. H0: There is no significant impact of market diversification and prices on Peru's mango exports in the period 2004 - 2024. 3. There is a significant impact of market diversification and prices on Colombia's mango exports in the period 2004 - 2024. H0: There is no significant impact of market diversification and prices on Colombia's mango exports in the period 2004-2024.

## 2. Methods

The present research, based on its study characteristics, was applied and with a quantitative approach. Correlational level, non-experimental design and hypothetical deductive method. It was applied because it sought, considering the results obtained in the initial research, to propose solutions to the social problems of countries through the formulation of problems and hypotheses.

It had a quantitative approach because research instruments and data collection of Peruvian and Colombian mango exports were used, as well as their statistical treatment to provide answers to the research questions.

Its scope was correlational as it sought to identify how the variables of market diversification, price and export were linked in the context of the Peruvian and Colombian mango industry.

It was of non-experimental design because the variables of market diversification, price and export were not subjected to any alteration and were studied without any manipulation.

Hypothetical deductive method, since it was based on a hypothesis of relationship between the study variables and had the purpose of ratifying or verifying this hypothesis, after a determined and objective procedure.

Longitudinal in section, because the dynamics of exports were studied annually, for 20 years.

As the population of this research, the numerical data consisting of the following indicators were taken into account: destination market numbers of Peruvian mangoes, FOB price of Peruvian mango exports, volume of Peruvian mango exported and FOB value of mango exported from both Peru and Colombia. In the same way, the research had a time limitation, since the data that was collected was from the period 2004 – 2024.

As for the variables in this study, three were taken into account. The first variable was market diversification. Market diversification could be defined as the strategy taken by organizations in order to expand their existing product offerings, but towards new geographical or demographic horizons ( Ferreira & Marques, 2024). This variable found the definition of its size in the number of target markets, being its main dimension. In this way, the number of countries to which Peru and Colombia exported the heading 08.04.50.20.00, during the years 2004 – 2024, were analyzed through Trade Map.

Another study variable of the research was price. It was represented in the value imposed on a commodity based on the capacity of a nation or company to produce a good, through economies of scale and monopolistic value ( Posada and Vélez, 2008). In the context of the research that was based on exports, the dimension that allowed the measurement of the variable was the FOB price. Through the Trade Map database, figures in dollars of the FOB unit price of mangoes, expressed in US dollars per kilogram, were collected.

Finally, the export variable was taken into account. It could be understood as the commercial exchange of a country that has an abundance of resources and that has the capacity to compete in the global market ( Páez et al., 2021). To dimension this variable in the context of the study, the volume of the exported product and its FOB value were taken into account. These dimensions were also extracted from Trade Map, where the total volume of Peruvian and Colombian mangoes exported was expressed in kilograms and the FOB value was expressed in thousands of US dollars.

The research population was the universe of data extracted according to the dimensions (number of markets, FOB price, volume and FOB value). In this way, the sample is based on the period 2004-2024.

The inclusion criteria of this study considered only mango exports from Peru and Colombia, as well as exports made in the period 2004-2024. In this way, mango exports from other countries were excluded, as well as Peruvian and Colombian exports that were not within the determined period. Only tariff heading 08.04.50.20.00 (Mangoes and mangosteens, fresh or dried) was included.

The technique applied to the research was that of documentary analysis, having as a measurement instrument the documentary analysis sheet, which was a single one since the data were extracted from a single source (Trade Map).

The dataset can be found in the ZENODO repository, under the title "Dataset: Impact of market diversification and prices on mango exports in emerging economies in the period 2004-2024" ( Cosio et al., 2026), https://doi.org/10.5281/zenodo.18634017, License "Creative Commons Attribution 4.0 International".

## 3. Results

**Figure 1.**
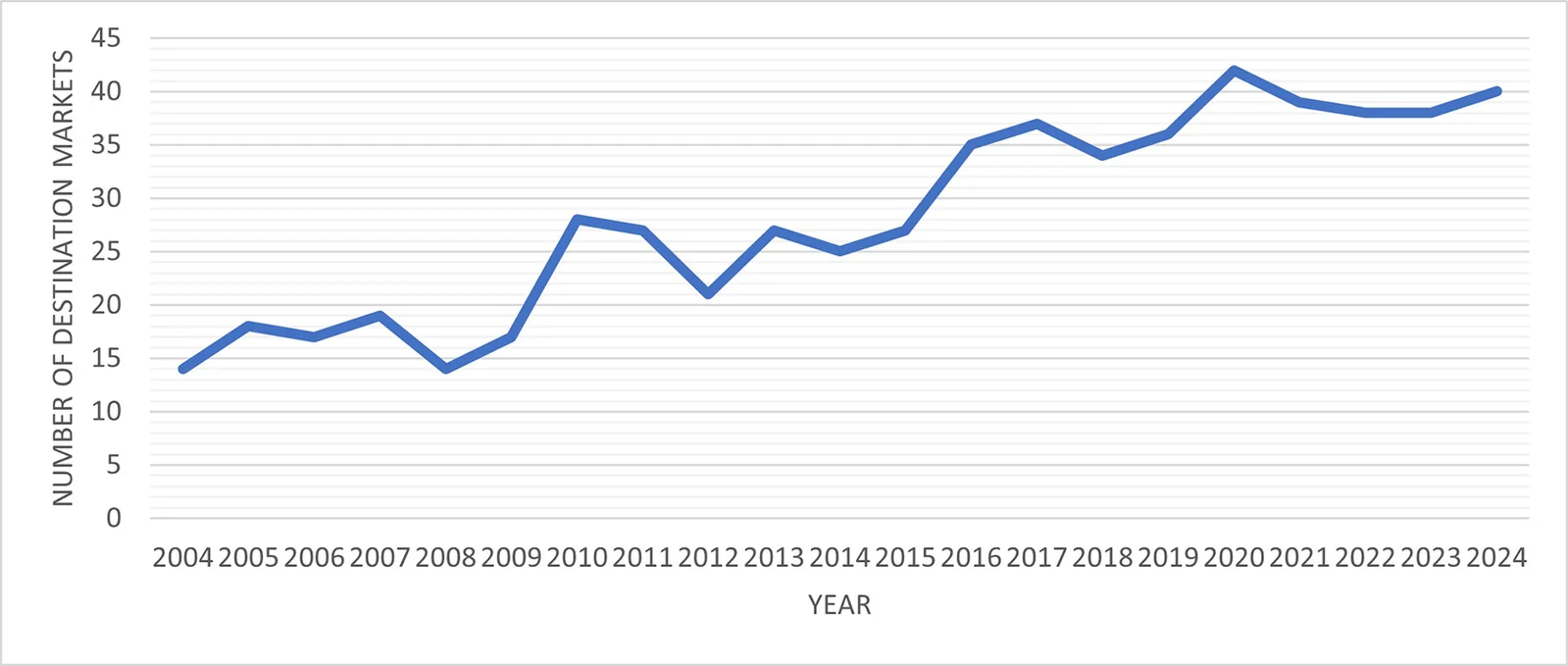
Number of destination markets for Peruvian mango exports, 2004-2024. Note. The graph represents the number of markets to which Peru exported heading 08.04.50.20.00, according to Trade Map.

Figure 1 showed that, throughout the period 2004-2024, the number of destination markets for Peruvian mangoes had an increasing performance, going from 14 markets in 2004 to 40 markets in 2024. During 2020, more markets were identified than last year, with the departure of Latvia, Lithuania, the Dominican Republic, Finland, Ireland, Australia, Sweden, Qatar and Aruba, as well as the entry of new markets such as Korea, Brazil, Colombia, Rancho de barcos y aeronaves, Uruguay and Portugal. During 2024, the main importer of Peruvian mangoes was the United States-

**Figure 2.**
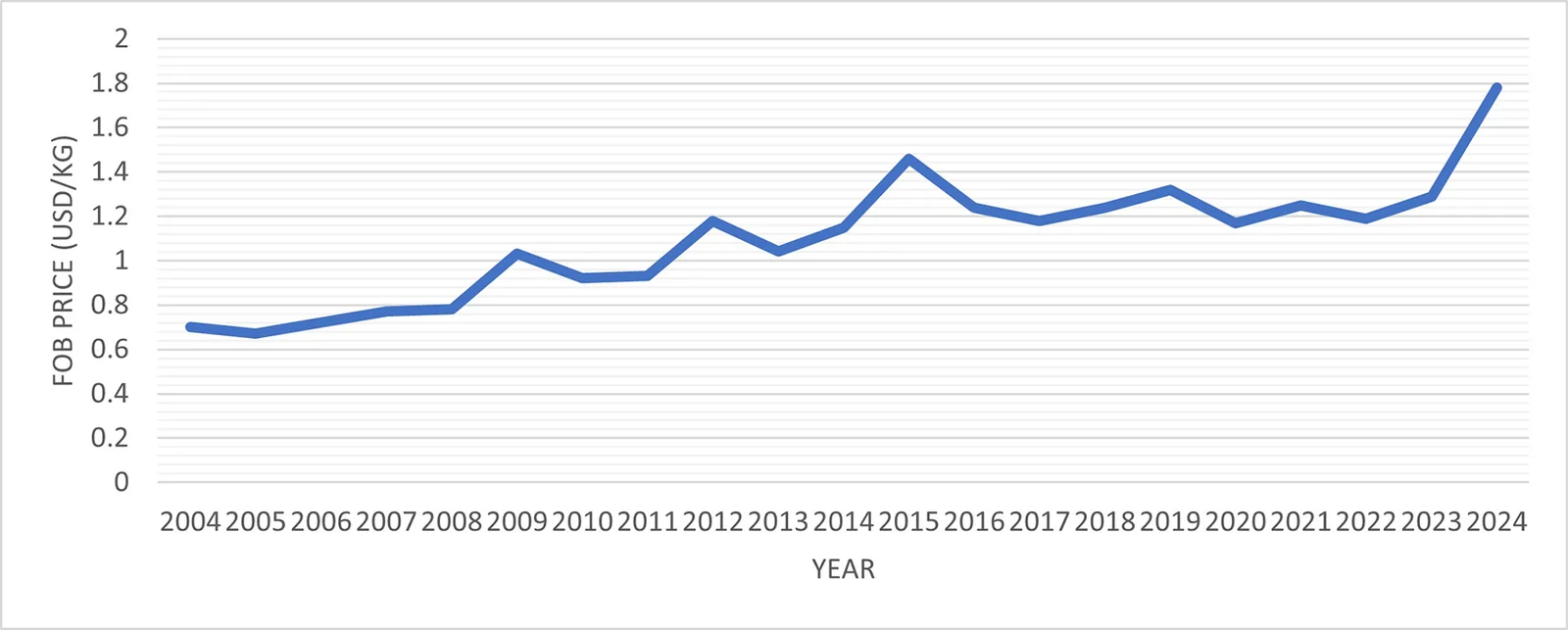
FOB price per kg of Peruvian mango exports, 2004-2024. Note. The graph represents the FOB unit price per kilogram exported+ or heading 08.04.50.20.00 of Peru, according to Trade Map.

According to figure 2, during the period 2004-2024, the FOB price per kilogram of Peruvian mango showed an increasing trend, with the exceptions of 2016, where it fell from USD1.46 in the previous year to USD1.24.

Similarly, in 2020 during the COVID-19 pandemic, the FOB price of a kilogram of Peruvian mango fell by USD0.15, growing little and having a fall again in 2022, recovering later. During 2024, the FOB price per kg of Peruvian mango was USD1.78. In general, the FOB price of mangoes showed constant growth and also showed a solid capacity for recovery, without such sharp falls. In the period studied, the FOB price did not show significant increases that could indicate price inflation, but neither did it show falls that showed a drastic depreciation.

**Figure 3.**
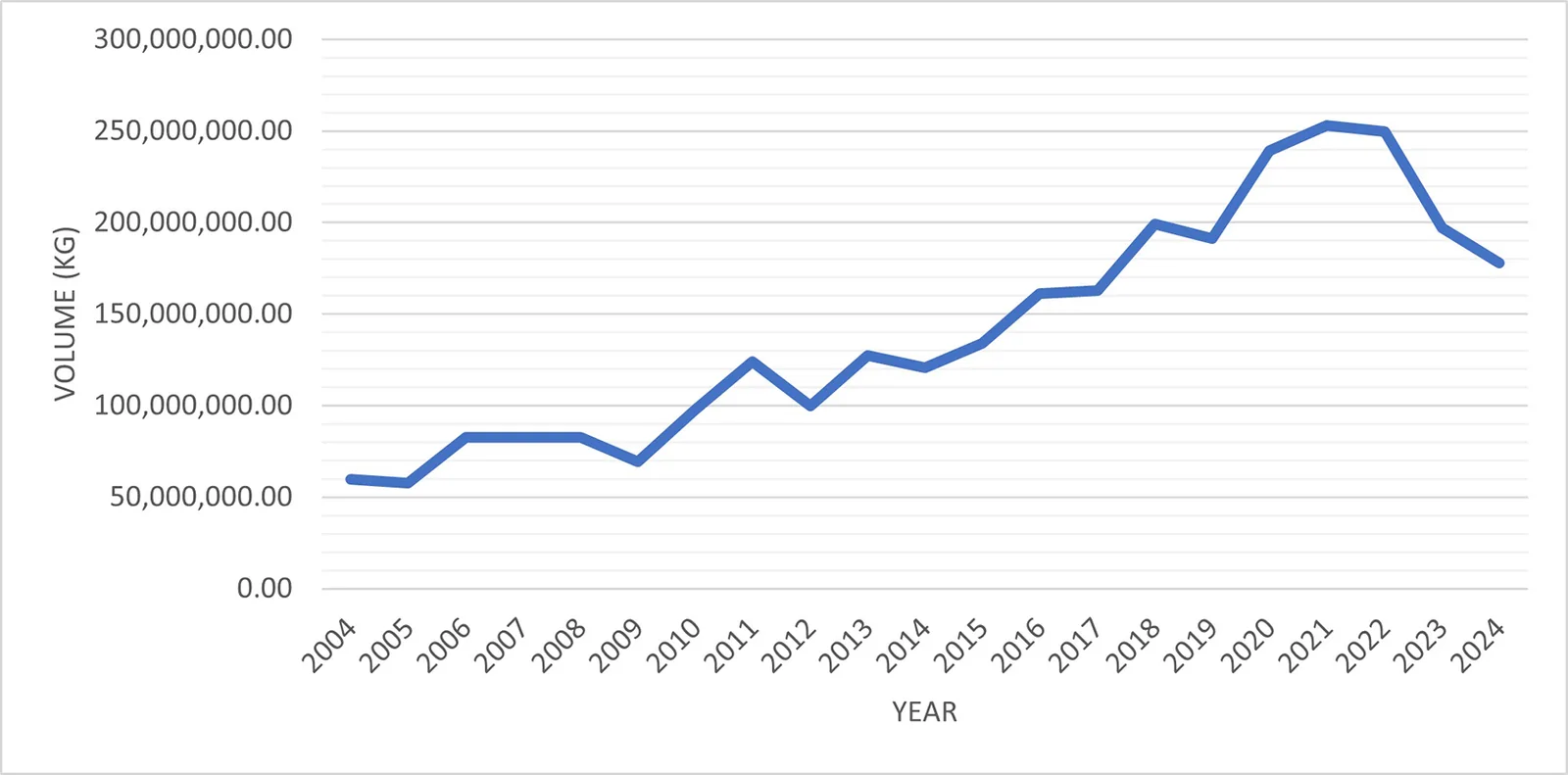
Volume (kg) of Peruvian mango exports, 2004-2024. Note. The graph represents the total volume, expressed in kilograms, exported by heading 08.04.50.20.00 from Peru, according to Trade Map.

Presenting the same trend, figure 3 showed how the volume of exports of heading 08.04.20.00 had a notorious and constant growth, except for the drop in volume during the period 2022-2024, due to the El Niño phenomenon that hit the coast of the country, where this fruit is mostly produced. In a general overview, the volume of Peruvian mango exports has shown stability and sustained growth throughout the study period, implying that Peruvian mangoes are increasingly consumed abroad.

**Figure 4.**
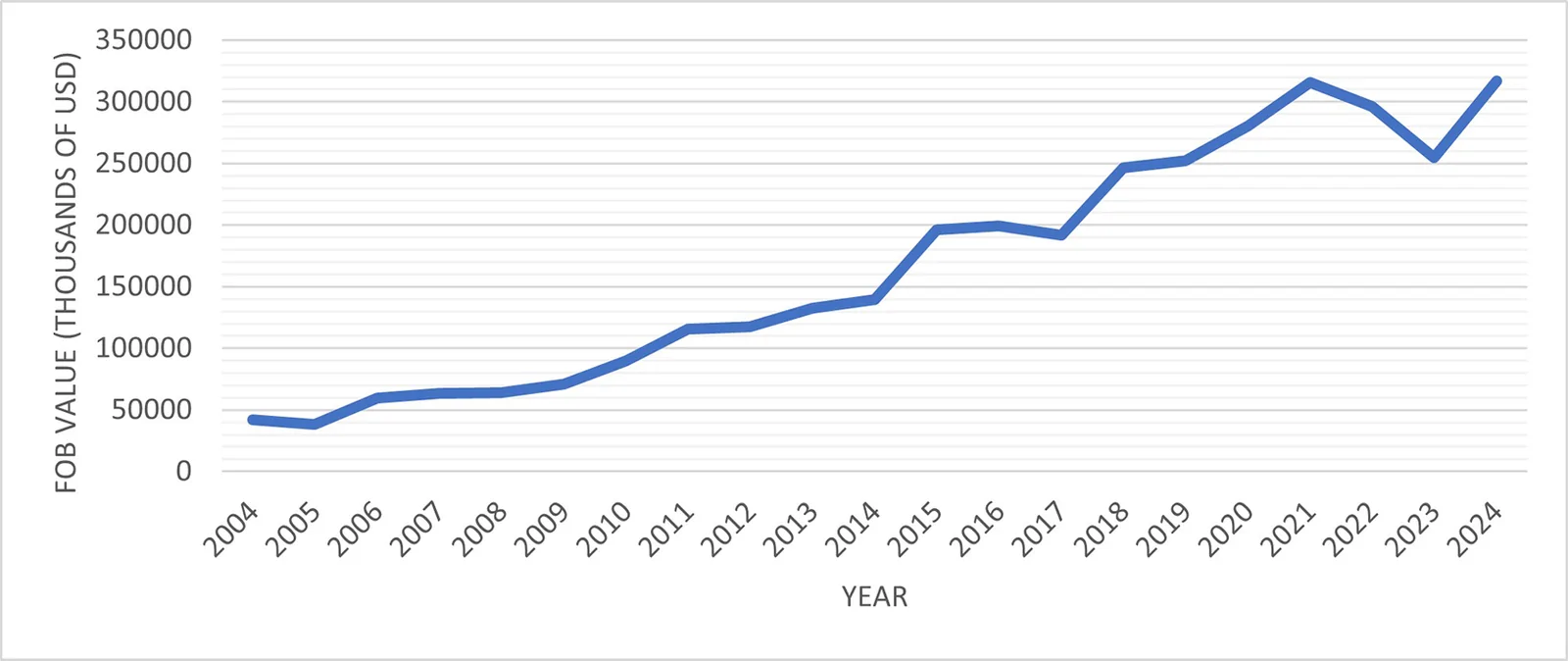
FOB value of Peruvian mango exports, 2004-2024. Note. The graph represents the total FOB value expressed in thousands of USD exported by the 08.04.50.20.00 item from Peru, according to Trade Map.

According to figure 4, the FOB value of Peruvian mangoes exported during the period 2004-2024 had a sustained growth performance, with a fall in 2017 of 7588 thousand USD and in 2023, with a fall of 41480 thousand USD. In the last year, exports heading 08.04.50.20.00 represented an FOB value of USD 316986 thousand.

**Figure 5.**
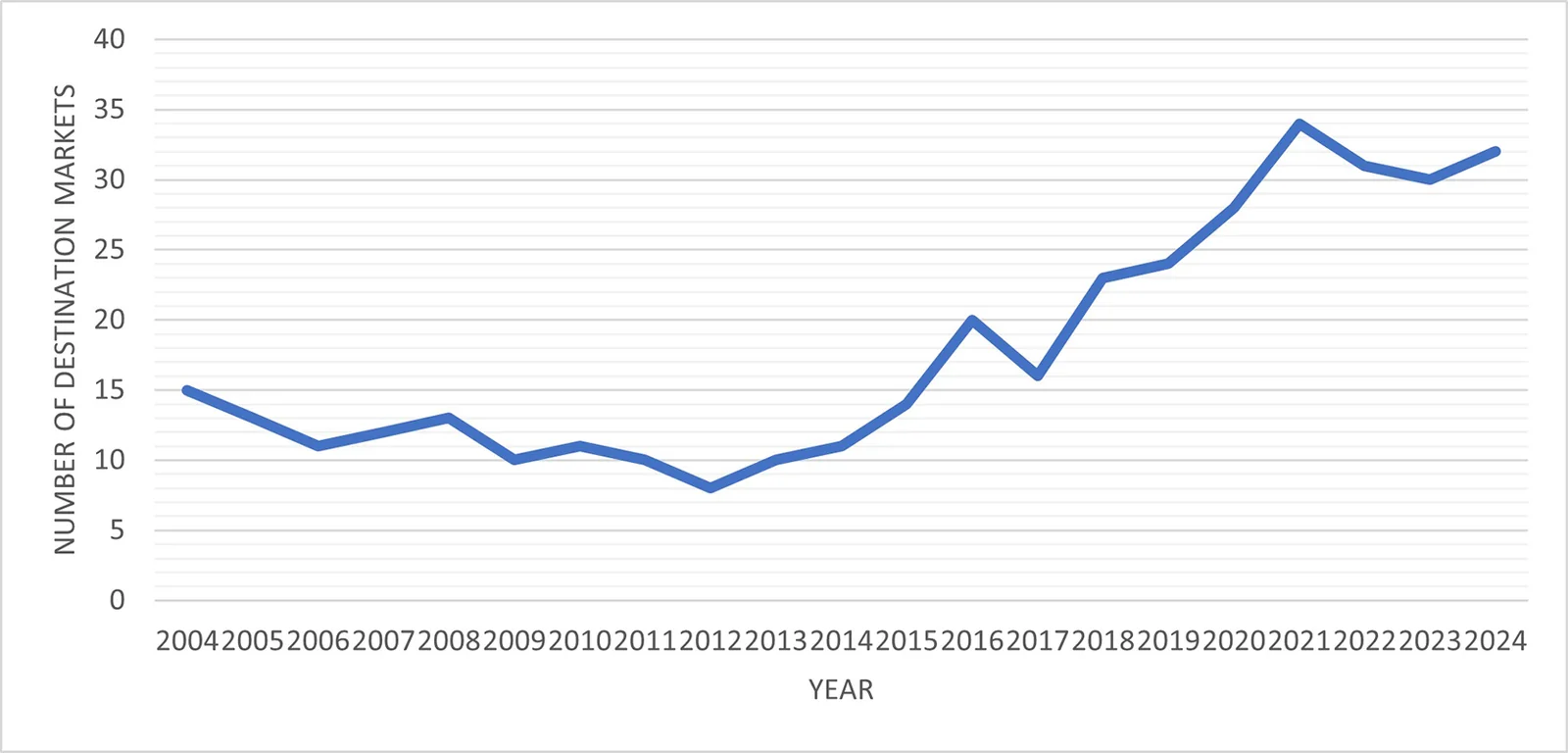
Number of destination markets for Colombian mango exports, 2004-2024. Note. The graph represents the number of markets to which Colombia exported the heading 08.04.50.20.00, according to Trade Map.

Regarding the dynamics of the destination markets for Colombian mangoes, figure 5 showed that, throughout the period studied, this fruit experienced sustained growth, going from 15 markets in 2004 to 32 markets in 2024. This growth was especially boosted from 2016, when it went from 14 to 20 countries to which mangoes were exported.

Despite the COVID-19 pandemic in 2020, there was no drop in the number of markets, but rather increased from 24 to 28 countries. The year with the largest number of destination markets was 2021, with 34 countries.

**Figure 6.**
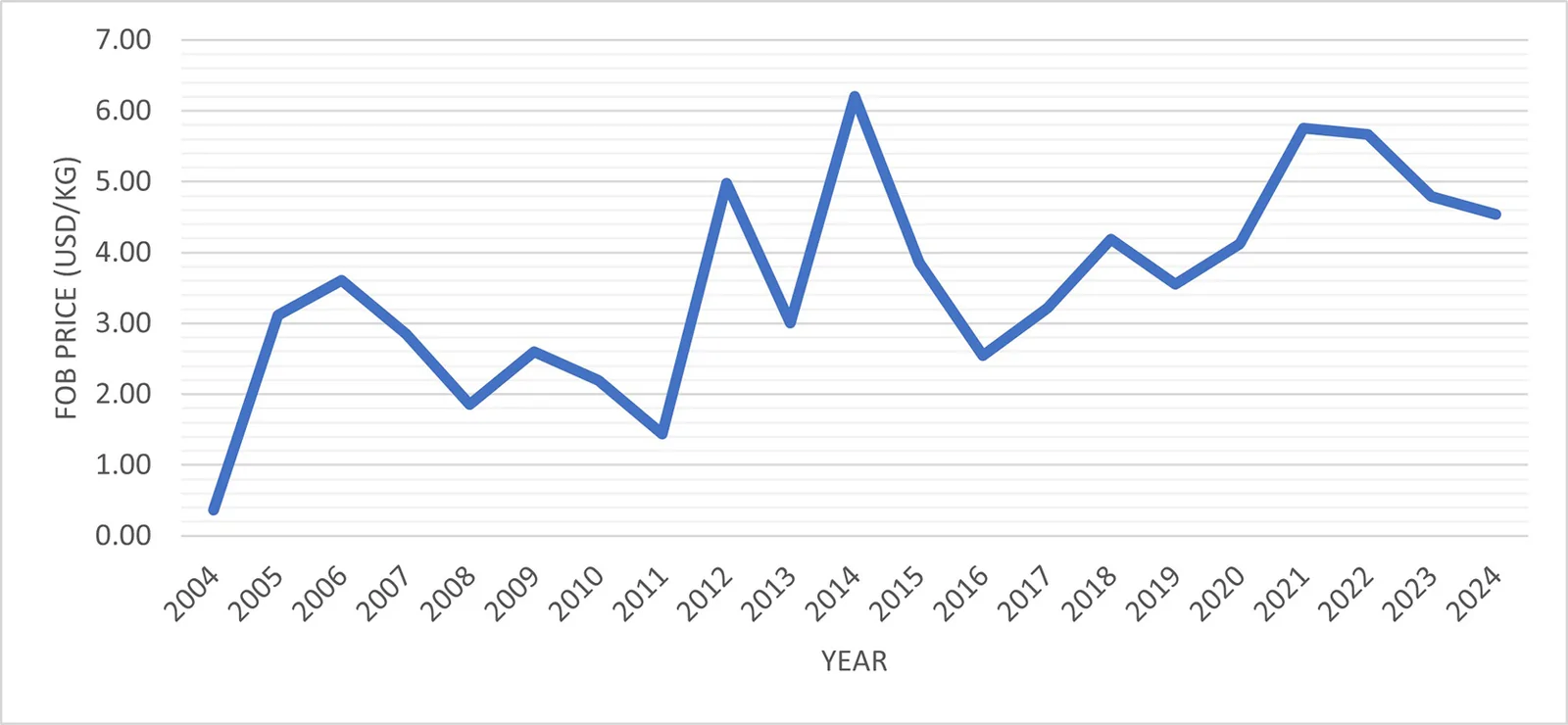
FOB price of Colombian mango exports (2004-2024). Note. The graph represents the FOB unit price per kilogram exported+ or heading 08.04.50.20.00 from Colombia, according to Trade Map.

According to figure 6, during the period 2004-2024, the FOB price per kilogram of Colombian mango showed a volatile and growing trend. Compared to the mango trend, which was more stable, the prices of the Colombian product presented constant falls and recoveries. It went from USD 0.36 in 2004 to USD 6.21 in 2014, this being its highest price of the entire period considered. Despite the fact that prices showed stability in the last decade, there was a decline in the 2022-2024 period.

**Figure 7.**
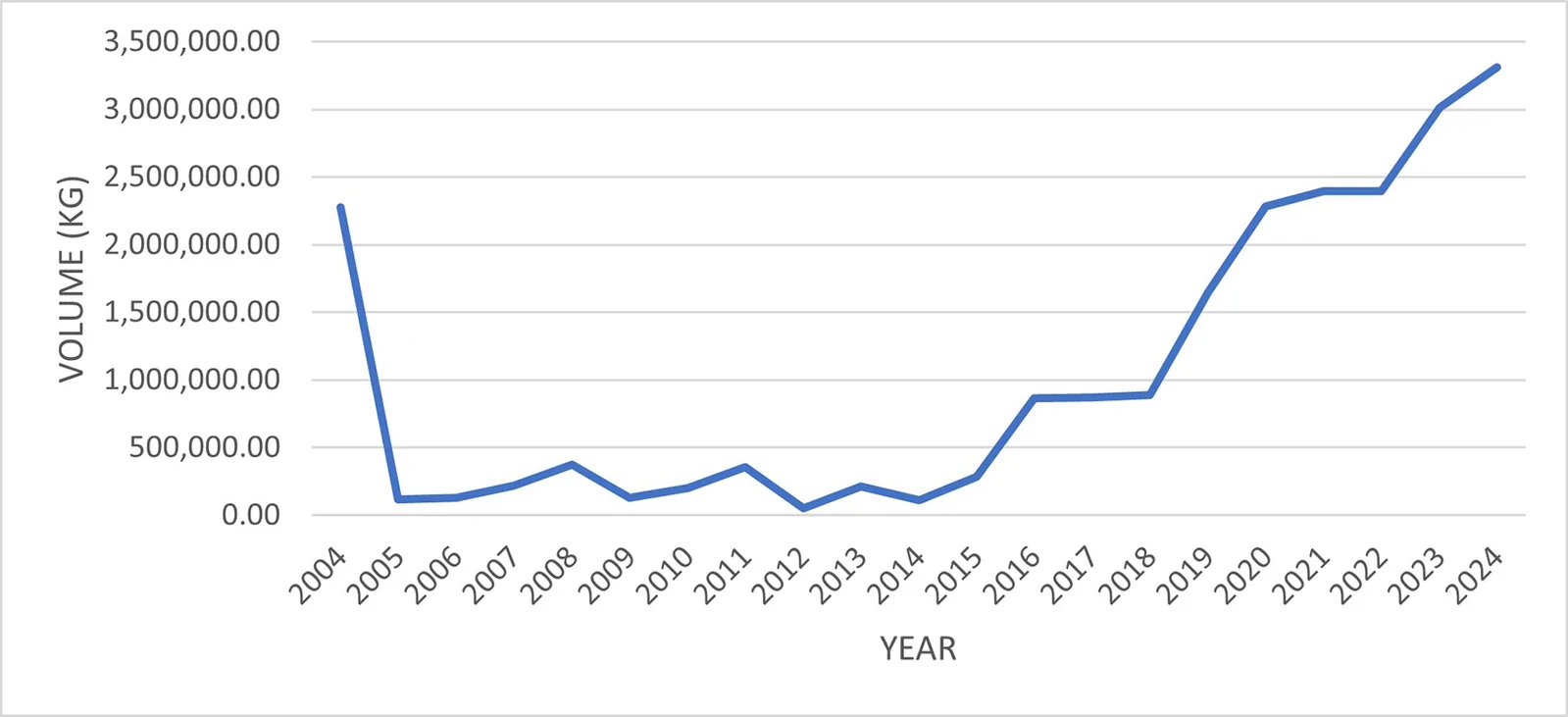
Colombian mango export volume, 2004-2024. Note. The graph represents the total volume, expressed in kilograms, exported by heading 08.04.50.20.00 from Colombia, according to Trade Map.

Figure 7 showed that, in the volume of Colombian mango exports, a stable growth dynamic was also observed, especially from 2015, where it went from 283,270 kg to 864,451 kg, its highest volume exported since 2005. In the last year, the volume of Colombian mango exported reached its highest peak within the period studied, with 3,312,224 kg exported. Broadly speaking, the volume of exports did not reflect significant drops, which shows that Colombian mangoes grew more and more in foreign consumption.

**Figure 8.**
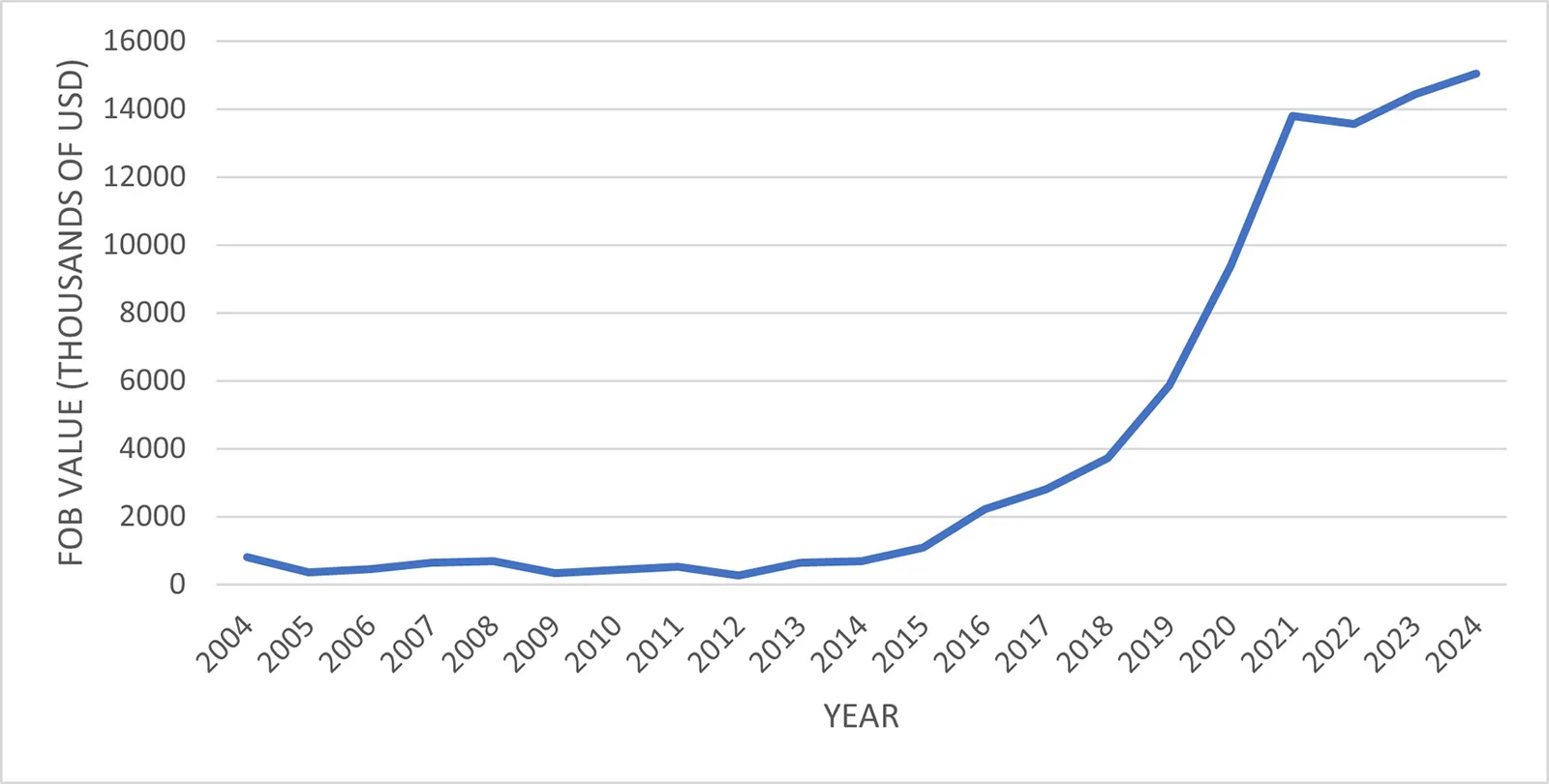
FOB value of Colombian mango exports, 2004-2024. Note. The graph represents the total FOB value expressed in thousands of USD exported by the 08.04.50.20.00 item from Peru, according to Trade Map.

A scenario like the other dimensions was shown in figure 8. The FOB value of Colombian mangoes in the two decades studied. It was observed that, although in the first ten years there was no growth of great magnitude, from 2015 the FOB value of exports skyrocketed, going from 1093 thousand USD in 2015 to 13,787 thousand USD by 2021. The COVID-19 pandemic did not generate a negative impact either, as the FOB value was resilient and growing. In 2024, the highest FOB value of mangoes during the entire study period was presented with 15,037 thousand USD.

Regarding specific hypothesis 1, which suggests that there is a significant relationship between market diversification and the prices of mango exports from Peru and Colombia in the period 2004-2024, the Pearson correlation coefficient that was calculated showed that the relationship between the dimension number of destination markets and FOB price was 0.756; that is, there was a high positive relationship between both dimensions, according to Table 1. Likewise, it was observed in the table that Sig. <0.001, so it could be stated that there was a significant relationship between the dimensions analyzed.

**Table 1.** Relationship between market diversification, prices and exports of Peruvian mango in the period 2004-2024.

		NUMBER OF DESTINATION MARKETS	FOB PRICE (USD/KG)	VOLUME (KG)	FOB VALUE (THOUSANDS OF USD)
NUMBER OF DESTINATION MARKETS	Pearson correlation	1	.756	.925	.937
	Sig. (bilateral)		<.001	<.001	<.001
	N	21	21	21	21
FOB PRICE (USD/KG)	Pearson correlation	.756 ^***^	1	.664 ^**^	.837 ^***^
	Sig. (bilateral)	<,001		.001	<,001
	N	21	21	21	21
VOLUME (KG)	Pearson correlation	.925 ^***^	.664 ^**^	1	.957 ^***^
	Sig. (bilateral)	<,001	.001		<,001
	N	21	21	21	21
FOB VALUE (THOUSANDS OF USD)	Pearson correlation	.937 ^***^	.837 ^***^	.957 ^***^	1
	Sig. (bilateral)	<,001	<,001	<,001	
	N	21	21	21	21

*** Correlation at 0.001(2-tailed)

** The correlation is significant at the 0.01 level (bilateral).

This explained that, as Peruvian mangoes managed to penetrate a greater number of markets, an increasing effect was generated applied to the unit price (per kg). In this way, it was understood that geographical amplification not only acted to give greater visibility to Peruvian mangoes but also boosted their valuation in markets that were likely to seek quality, certifications, or differentiation, thus allowing exporters to also increase their profitability through higher prices ( MIDAGRI, 2022).

The results also showed that the ratio between the number of destination markets and volume was 0.925; that is, there was a very high positive correlation between both dimensions. In the same way, it was observed that Sig. <0.001, so it could be stated that there is a significant relationship between the dimensions.

The correlation of the dimensions reflected that the presence of Peruvian mangoes in more countries was accompanied by a key growth in the number of mangoes that were placed abroad. This dynamic was explained by the fact that, logically, diversification facilitated the absorption of greater volumes, thus increasing the stability of Peruvian exports in the face of fluctuations or crises that some of the destination markets may have faced.

Regarding the correlation between the number of destination markets and the FOB value exported, it was shown as a result that the Pearson correlation was 0.937; that is, there was a very high positive relationship between both dimensions. The results also showed that Sig. <0.001, so it could be stated that there was a significant relationship between the dimensions.

The relationship between the dimensions revealed that, by expanding to a greater number of international markets, Peruvian mangoes considerably increased the total value of their sales abroad. This behavior suggested that the entry into new destinations not only increased the quantity exported, but also facilitated the generation of greater economic income, thus reflecting a better exploitation of commercial opportunities and a greater ability to capture value in the global market.

By having a larger portfolio of customers from other nations due to the demand for healthy foods and Peru's ability to meet the needs that its domestic markets could not meet on the same scale ( MIDAGRI, 2022), dollar revenues were diversified. This dynamic highlighted the importance of diversification strategies as fundamental factors for the sustainable growth and profitability of the mango export sector in Peru.

Following this line, the Pearson correlation was used to answer the relationship between prices and exports of Peruvian mangoes. The results showed that the ratio between FOB price and volume was 0.664; that is, there was a moderate positive relationship between both dimensions. Although the relationship was not as strong as the other relationships analyzed, the statement was still maintained that as the FOB price of mangoes rose, so did the volume or quantity in kilograms exported. Similarly, Table 8 showed that Sig. 0.001, so it could be stated that there was a significant correlation between the dimensions.

This meant that, when FOB prices increased, the volume exported followed the same trend. This suggested that, in a market with sufficient supply and stable demand, prices and volumes behaved in a moderate alignment. The increase in prices did not necessarily limit the quantity exported, but, in this context, both indicators grew in parallel, probably due to the high competitiveness and quality of Peruvian mangoes, factors that favored the expansion of supply in international markets without reducing demand.

Pearson's correlation between FOB price and FOB value showed a result of 0.837; that is, there was a high positive relationship between the dimensions studied. Table 9 also showed that Sig. <0.001, thus demonstrating that there was a significant correlation between the dimensions.

This meant that, as the average price per kg exported increased, the total value obtained by exports also grew. The logic of this correlation was direct: the total FOB value was composed of the quantity exported multiplied by the unit price, so the increase in price had a multiplier effect on total income, because there was no considerable decrease in volume. Thus, the increase in the FOB price was reflected in a higher monetary value of exports, indicating that improvements in unit prices effectively contributed to the increase in income for the mango export sector during the study period.

The Pearson correlation was calculated for the dimensions focused on Colombian mango exports.

According to Table 2, the correlation between the number of destination markets and the FOB price was 0.497; that is, there was a moderate positive correlation between the dimensions analyzed. Likewise, a significant correlation was shown, as the results showed that Sig. <0.022. The above explained that as the number of destination markets for Colombian mango exports expands, so did the FOB price to a moderate extent. The expansion of the Colombian mango export offer to new geographical destinations allowed the country to maintain competitive prices as it is a product with a greater positive perception of the quality of Colombian mango.

**Table 2.** Relationship between market diversification, prices and exports of Colombian mango in the period 2004-2024.

		NUMBER OF DESTINATION MARKETS	FOB PRICE (USD/KG)	VOLUME (KG)	FOB VALUE (THOUSANDS OF USD)
NUMBER OF DESTINATION MARKETS	Pearson correlation	1	,497 ^*^	,900 ^**^	,951 ^**^
	Sig. (bilateral)		0.022	0.000	0.000
	N	21	21	21	21
FOB PRICE (USD/KG)	Pearson correlation	,497 ^*^	1	0.284	,572 ^**^
	Sig. (bilateral)	0.022		0.212	0.007
	N	21	21	21	21
VOLUME (KG)	Pearson correlation	,900 ^**^	0.284	1	,903 ^**^
	Sig. (bilateral)	0.000	0.212		0.000
	N	21	21	21	21
FOB VALUE (THOUSANDS OF USD)	Pearson correlation	,951 ^**^	,572 ^**^	,903 ^**^	1
	Sig. (bilateral)	0.000	0.007	0.000	
	N	21	21	21	21

** The correlation is significant at the 0.01 level (bilateral).

* The correlation is significant at the 0.05 level (bilateral).

On the other hand, the correlation applied to the number of destination markets and volume resulted in 0.900, which reflected the existence of a very high positive correlation between the dimensions, as well as a strong significance, since Sig. <0.000. These results reflected that, as the destination markets for Colombian mango exports expanded, the country's capacity to market a higher quantity of kg of mango expanded, thus contributing to the stability of exports of the fruit by expanding its supply in quantities.

When reviewing the relationship between the number of markets and the total FOB value exported, a statistically very high correlation was observed (coefficient of 0.951; Sig.=0.000). Total mango export revenue increased sharply as the portfolio of international destinations expanded. The results indicated that each new market generated opportunities to increase both volume and income in dollars, strengthening the ability of Colombian exporters to take advantage of trade advantages and capture value in the global market. This dynamic drove improvements in business performance and directly contributed to the economic sustainability of the sector.

The ratio of FOB price to exported volume was low and negligible (0.284; Sig<0.212) in this context, suggesting that price increases were not necessarily accompanied by higher volumes. However, the link between FOB price and FOB value was stronger and more relevant (coefficient of 0.572; Sig. <0.007): total revenues grew each time the unit price increased. This dynamic, as in the case of Peru, showed that when there was an increase in prices, the total value of exports behaved following the same trend. This was logical due to the multiplier effect that the FOB price has on the total monetary income of Colombian mango exports.

Having analyzed the results of the correlation, the alternate hypothesis (Ha) could be accepted, and the null hypothesis (H0) is rejected.

With regard to specific scenario 2, which indicates that there is a significant influence of market diversification and prices on Peru's mango exports in the period 2004-2024, it was shown in Table 3 that the statistical analysis examined in detail how market diversification and the FOB price per kilogram jointly influenced the total value of exports, represented by the FOB Value. The results revealed an exceptionally high multiple correlation of 0.957, which meant that these two variables explained 90.7% of the total variation in the FOB Value. In essence, almost all of the fluctuations observed in the value of Peruvian exports were due to the extension of destination markets and the unit price of products sent abroad.

**Table 3.** Summary of the multiple linear regression model applied to Peru.

Model	R	R squared	R square adjusted	Standard Estimate Error
2	,957b	0.917	0.907	29283.398

^a^ Predictors: (Constant), Market diversification

^b^ Predictors: (Constant), Market Diversification, FOB Price (USD/KG)

According to Table 4, the ANOVA test validated the vigor of the complete model, registering an F statistic of 99.001 and a p-significance value of 0.000, much lower than the conventional threshold of 0.05. This confirmed that the set of predictors far exceeded any random or simple average-based explanations, demonstrating a statistically robust and reliable influence on the FOB Value.

**Table 4.** ANOVA test applied to Peru.

Model		Sum of squares	Gl	Quadratic mean	F	Gis.
2	Regression	169790908284.402	2	84895454142.201	99.001	,000b
	Residue	15435312846.264	18	857517380.348		
	Total	185226221130.667	20			

^a^ Dependent variable: FOB value (Thousands of USD)

^b^ Predictors: (Constant), Market Diversification, FOB Price (USD/KG)

In the individual coefficients, Table 5 showed how the constant was established at -153.787.268 with a t of -5.768 and p of 0.000, acting as a theoretical intercept that indicated the hypothetical FOB value in the total absence of diversification and price, an unrealistic situation in the Peruvian export context. Market diversification showed a non-standardized coefficient of 7,269.197, a t of 6.830 and p of 0.000, with a standardized beta of 0.710, which implied that for each additional unit in diversification (each new Peruvian mango export market), the FOB Value increased by 7,269.197 thousand dollars, representing the dominant factor in the model. On the other hand, the FOB price (USD/kg) registered a coefficient of 104,375,426, t of 2,893 and p of 0.010, with a beta of 0.301, meaning that each extra dollar per kilo raised the FOB Value by 104,375,426 thousand dollars, a complementary but significant effect.

**Table 5.** Coefficients applied to Peru.

Model		Non-standardized coefficients		Standardized coefficients	t	Itself.
		B	Desv. Error	Beta		
2	(Constant)	-153787.268	26662.786		-5.768	0.000
	Market diversification	7269.197	1064.350	0.710	6.830	0.000
	FOB Price (USD/KG)	104375.426	36081.800	0.301	2.893	0.010

^a^ Dependent variable: FOB value (Thousands of USD).

In this way, the linear regression equation applied to know the influence of market and price diversification on Peruvian mango exports was obtained:

$$Y_{\text{FOBvalue}}=-153787.27+104375.43\;x\;\mathrm{FOB}\;\text{Price}+7269.2\;x\;\text{Market diversification}$$

This equation combined a constant with coefficients associated with each variable, which allowed us to understand the individual and joint effect they had on the total value exported. The constant, which had a negative value, represented the theoretical value of the FOB value when there was no diversification or FOB price. Although this scenario is not realistic, it was useful as a reference point for the model. The coefficient associated with diversification showed that for each new market to which it was exported, the FOB Value increased around 7,269.2 thousand dollars, indicating a significant impact of opening new commercial destinations. The FOB Price coefficient reflected that, keeping diversification constant, each dollar increases in the price per kilogram generated an increase of 104.375 thousand dollars in the FOB Value. This underscored the importance not only of the number of markets, but also of the prices that were achieved in each one.

On the other hand, to obtain results against specific hypothesis 3 on the influence of market diversification and prices on Colombia's mango exports in the period 2004-2024, the linear regression analysis in Table 6 evaluated how market diversification predicted the FOB value of exports, first measuring the strength of its linear relationship using Pearson's correlation coefficient, which reached 0.951. This value, which was very high and positive (close to 1, where 1 represents a perfect correlation), indicated that both variables moved together almost flawlessly: the greater the diversification, the higher the FOB value, with a strong linear relationship according to statistical standards. The model was summarized in the adjusted R-squared of 0.899, which meant that 89.9% of the fluctuations in the FOB value were directly explained by market diversification, leaving only 10.1% for other factors.

**Table 6.** Summary of the multiple linear regression model applied to Colombia.

Model	R	R squared	R square adjusted	Standard Estimate Error
1	,951a	0.904	0.899	1732.342

^a^ Predictors: (Constant), Market diversification.

The ANOVA test in Table 7 confirmed the overall robustness of the model with an F statistic of 179.495 and a p-value of 0.000 (less than 0.001), demonstrating that the model far outperformed the simple average of FOB values and that the results were not the result of chance. This statistically validated that diversification acted as a real and powerful predictor in the FOB value of Colombian mango exports.

**Table 7.** ANOVA test applied to Colombia.

Model		Sum of squares	Gl	Quadratic mean	F	Itself.
1	Regression	538666215.777	1	538666215.777	179.495	,000b
	Residue	57019194.032	19	3001010.212		
	Total	595685409.810	20			

^a^ Dependent variable: FOB value (Thousands of USD)

^b^ Predictors: (Constant), Market diversification.

The results in Table 8 showed that the constant was located at -6,612.198, accompanied by a t-statistic of -7.433 and a p-statistical significance of 0.000. This value functioned mainly as a theoretical reference point, since it represents the assumed FOB Value in the total absence of diversification; However, in practice, exports never start from an environment without markets, so their interpretation serves above all to adjust the equation to historical data. On the other hand, the coefficient of the Market Diversification variable was 602.519, with a t of 13.398 and a p of 0.000, in addition to a standardized beta of 0.951. This indicated that for each additional market to which exports accessed, the FOB Value increased, on average, by 602,519 thousand dollars. The beta, very close to one, indicated that diversification was the dominant variable and the main explanation for the variations observed in the value exported.

**Table 8.** Coefficients applied to Colombia.

Model		Non-standardized coefficients		Standardized coefficients	t	Itself.
		B	Desv. Error	Beta		
1	(Constant)	-6612.198	889.539		-7.433	0.000
	Market diversification	602.519	44.972	0.951	13.398	0.000

^a^ Dependent variable: FOB value (Thousands of USD).

The high statistical significance of both coefficients (very low p-values) confirmed that the relationship found was hardly random, and that there is sufficient evidence to affirm that opening new markets brought tangible and quantifiable benefits to export performance. Thus, the alternative hypothesis (Ha) referring to the influence of market diversification on exports was accepted and the null hypothesis (H0) was rejected

In the regression analysis of Table 9, the influence of the variable "FOB price (USD/kg)" on the FOB value was evaluated, also considering market diversification. The system detected that the standardized coefficient for this variable would have been 0.131, which represents a small effect on the model. Likewise, the result of the t-test was 1.683 and the level of significance reached 0.110; Thus, the variable did not exceed the usual threshold of statistical significance required to justify its incorporation into the final model. On the other hand, the partial correlation stood at 0.369, which showed a moderate relationship between the FOB price and the FOB value, after controlling for market diversification. However, this relationship was not strong enough or significant according to the statistical criteria used during the analysis. As a consequence, SPSS classified it in the "Excluded variables" section, evidencing that its contribution was insufficient from a statistical point of view and did not contribute significantly to the explanation of the model obtained. Therefore, the alternative hypothesis (Ha) referring to the influence of market diversification and Colombian mango prices in the period studied was partially accepted, and the null hypothesis (H0) is rejected.

**Table 9.** Variables excluded in the linear regression model applied to Colombia.

Model		In beta	t	Gis.	Partial correlation	Cocollinearity Statistics
						Tolerance
1	FOB Price (USD/KG)	.301b	2.893	0.010	0.563	0.429

^a^ Dependent variable: FOB value (Thousands of USD)

^b^ Predictors in the model: (Constant), Market diversification

As in the case of Peru, the linear regression equation applied to know the influence of market diversification and price on Colombian mango exports was obtained:

$$Y_{\text{FOBvalue}}=-6612.2+602.52\;x\;\text{Market diversification}$$

The estimated linear regression model indicated that the FOB value of exports depended on the degree of market diversification. The results indicated that, for the period analyzed, an increase of one unit in the market diversification index was associated with an increase of 602.52 monetary units in the FOB value exported. The constant of −6612.2 represented the theoretical level of the FOB value when the diversification index was equal to zero.

## 4. Discussion

In this research, whose general objective was to evaluate the impact of market diversification and prices on mango exports from Peru and Colombia in the period 2004–2024, it was observed that both variables were closely linked to export performance, although in a differentiated way depending on the country. The statistical results showed that market diversification was systematically associated with growth in the volume and FOB value exported, while the effect of prices was clearer in the case of Peru than in Colombia, which led to qualify the acceptance of the hypothesis raised. This dynamic was interpreted from the international trade and competitive advantage approaches that explained how specialization, factor endowments, and business strategies combined to drive the external insertion of mangoes. This behavior was understood from the logic of international trade exposed by Ricardo ( López, 2012), where specialization in goods with relative advantages made it possible to transform productive capacities into greater external income.

In relation to the first specific objective, which was to identify the relationship between market diversification and mango prices from Peru and Colombia, the results showed that when mangoes reached more countries, the FOB price tended to improve. This indicated that diversification not only opened doors to new buyers, but also brought closer markets with greater purchasing power and more demanding quality standards, which contributed to a better valuation of the product. This finding was connected to what was described by Montes et al. (2024) in the case of blueberries, where excessive dependence on a few markets generated vulnerabilities, while openness to new destinations was associated with higher levels of competitiveness. It also coincided with what García et al. (2025) identified for Colombian coffee: the lower the concentration of markets, the greater the capacity to sustain and improve the value exported.

From the theory, this relationship was explained with Ansoff's product-market matrix taken up by Ferreira and Marques (2024), which proposed expansion into new markets as a growth strategy for already consolidated products. In the mango market, this logic was reflected in the passage from a few to many destinations, which allowed the product to be positioned in niches willing to pay more, in exchange for quality and differentiated attributes. The gradual internationalization model of Johanson and Wiedersheim (1975) helped to understand that this expansion was not improvised, but a process of progressive learning from closer markets to more complex ones, which made it possible to reduce errors and better adjust pricing strategies. Added to this was the model of Hitt et al. (1997), who warned that diversifying geographically implied assuming higher costs and management challenges, but also offered clear benefits in terms of knowledge, negotiation and risk reduction, as reflected in the evolution of FOB prices without significant deterioration in demand.

Regarding the second specific objective, which was to evaluate the impact of market diversification and prices on Peru's mango exports in the period 2004–2024, the results showed that the growth in the number of destinations had a clearly positive effect on the volume and FOB value exported. In Peru, the increase from 14 to 40 destination markets resulted in a notable increase in the volume exported and the FOB value.

This impact of diversification on exports was interpreted under the theory of international trade and the factor proportion model developed by Krugman ( Páez et al., 2021), according to which factor endowments explained export specialization in natural resource and labor-intensive products, such as mangoes. The abundance of suitable land, favorable climatic conditions and availability of labor allowed Peru to sustain a growing supply to supply increasingly numerous markets, consistent with the explanation of these authors on the link between factor endowment and trade pattern

Regarding the impact of prices, the correlations between the FOB price, the volume exported and, above all, the FOB value were positive and statistically significant, which meant that price increases did not discourage the quantity exported and did translate into higher revenues for the sector. This dynamic was reminiscent of what happened with quinoa, where Huacani et al. (2024) showed that the link between export prices and quantities was significant and that the combination of promotion strategies and international positioning made it possible to sustain demand despite price variations. From the theoretical basis, the trade and price framework worked on by Krugman ( Páez et al., 2021), the monopolistic competition and economies of scale approach discussed by Posada and Vélez (2008) and the export price formation model proposed by Baena and Cano (2022) helped to understand why Peru managed to transform price increases into higher revenues without losing demand. In this country, the combination of cost advantages, product differentiation, and a relatively manageable logistics cost structure allowed the increase in the FOB price to reinforce the value exported.

In the third specific objective, aimed at evaluating the impact of market diversification and prices on Colombia's mango exports between 2004 and 2024, it was observed that the expansion from 15 to 32 destinations was accompanied by a significant jump in both dimensions from the mid-2010s onwards. This trajectory was consistent with what was pointed out by Arana et al. (2021) in ginger, where the management of internal factors and orientation to external markets allowed for improved export performance, and with the results of Cubillos et al. (2021), which showed how palm oil steadily increased its share of agricultural exports to the European Union after the entry into force of the trade agreement. Similarly, the evidence from Huacani et al. (2024) in quinoa indicated that the articulation between trade policy and external promotion was key to enhancing the international presence of an agricultural product, which offered a parallel with the behavior of mangoes.

This is supported by the theory of competitive advantage put forward by Michael Porter ( Páez et al., 2021) when he stressed that the provision of resources was not enough, but that it was necessary to consolidate improvements in quality, processes, infrastructure, and differentiation so that market diversification could effectively be transformed into higher export revenues. Along these lines, the experience reported in products such as coffee ( García et al., 2025), avocado ( Torres & Trochez, 2023) and palm oil ( Cubillos et al., 2021) showed that positioning and reputation-building strategies were decisive in fully taking advantage of the opportunities opened up by geographical diversification.

As for the price, on the other hand, the behavior was more unstable and less clear. The series of prices of Colombian mangoes presented greater volatility and these changes were not always reflected proportionally in the volume or FOB value exported, which suggested that other elements, such as the scale of production, logistical conditions or the configuration of the destination markets, had a more determining role than the price itself. Therefore, it was an excluded variable in the multiple linear regression model. Based on this evidence, it was concluded that the hypothesis about the impact of market diversification and prices on Colombian mango exports was only partially accepted: it was only true in the case of the market diversification variable, but it was not verified with the same force for the price variable.

In Colombia, the lower consolidation of mangoes as an emblematic product, greater price volatility and possible logistics and market cost pressures limited the price's ability to boost exports by the same magnitude as Peru.

## 5. Conclusions

It was concluded that there is a significant impact of market diversification and prices on mango exports from Peru and Colombia in the period 2004-2024. The changes in the number of destination markets and in the FOB price were reflected in relevant variations in the volume and value exported, which confirms that these variables not only accompany, but also condition the performance of the sector in the period studied, although not with the same force in both cases. In general terms, expanding the list of destinations made it possible to sustain and increase the volume and FOB value exported, while prices clearly contributed to the growth of Peruvian exports, but had a more limited effect on Colombia.

In addition, it was concluded that there is a significant relationship between market diversification and mango prices in Peru and Colombia in the period 2004–2024. When looking at the relationship between diversification and prices, it was seen that the more markets expanded, the better price conditions they tended to achieve. In Peru, the correlation between the number of markets and FOB price was high and significant (r = 0.756; Sig. = 0.001), suggesting that reaching more destinations allowed positioning itself in niches willing to pay better for mangoes. In Colombia, the relationship was more moderate, but also significant (r = 0.497; sig. = 0.022).

It was also concluded that there is a significant impact of market diversification and prices on Peru's mango exports in the period 2004-2024. The regression model shows that diversification had a very marked effect on the value exported (Beta = 0.710; B = 7,269,197; Sig. = 0.000). In practice, every time Peruvian mangoes entered new destinations, space was opened to place more volume and generate more income, while the regression model shows that the price clearly contributes to explaining the value exported (Beta = 0.301; B = 104,375,426; Sig. = 0.010), which indicates that not only was more exported, but also better paid.

Finally, it was concluded that there is a partial significant influence of market diversification and prices on Colombia's mango exports in the period 2004-2024. The final regression model shows that diversification was the key factor (Beta = 0.951; B = 602,519; Sig. = 0.000), which meant that the jump from a few markets to more than thirty was decisive for Colombian mango exports to take off.

On the other hand, the price did not end up consolidating itself as a decisive factor. In the regression model, the price did not reach significance (Beta ≈ 0.131; Sig. = 0.110), so it was left out of the final model. In Colombia, exports grew mainly because more markets were reached, not because the price has been a driver, which is why the objective set is only considered fulfilled for the diversification of markets.

## Data Availability

ZENODO repository. Dataset: Impact of market diversification and prices on mango exports in emerging economies in the period 2004-2024. https://doi.org/10.5281/zenodo.18634017 ( Cosio et al., 2026). This project contains the following underlying data:
• DATASET. (It contains a database of the number of destination markets, FOB price, volume, and FOB value of mango exports from Peru and Colombia). • CORRELATIONS - COLOMBIA. (It contains the statistical values of the correlations of the study variables for the case of Colombia). • CORRELATIONS - PERU. (It contains the statistical values of the correlations of the study variables for the case of Peru). • MULTIPLE LINEAR REGRESSION MODEL COLOMBIA. (It contains the values of the multiple regression model for Colombia). • MULTIPLE LINEAR REGRESSION MODEL PERU. (It contains the values of the multiple regression model for Peru). Data is available under the terms of the license “ Creative Commons Attribution 4.0 International”.
