# Market Diversification and Mango Exports in Emerging Economies: Evidence from Peru and Colombia, 2004-2024

**DOI:** 10.12688/f1000research.177965.2

**Published:** 2026-06-23

**Authors:** Ricardo Fernando Cosio Borda, Janel Janelvia Ancajima Velásquez, Percy Hugo Quispe-Farfán, Natalie Margarita Gamarra-Vargas, Esther Rosa Saenz Arenas, Omar Alonso Patiño Castro, Berenice Cajavilca-Gonzáles, Jorge Luis Cardich Pulgar

**Affiliations:** 1 Facultad de Administración, Finanzas y Ciencias Económicas, Universidad EAN, Bogotá, Colombia; 2 Universidad César Vallejo, Piura, Peru; 3 Universidad Peruana de Ciencias Aplicadas, Lima, Peru; 4 Universidad Tecnológica del Perú, Lima, Peru; 5 Universidad San Ignacio de Loyola, Lima, Peru

**Keywords:** Agroindustry, exports, diversification, market, price.

## Abstract

Within global agri-food trade, mango has become a tropical fruit of strategic relevance. However, export growth is sustainable only when it is supported by diversified markets, competitive stability, and access to destinations with effective purchasing capacity. This study examines market diversification and mango export competitiveness in Peru and Colombia, two emerging economies, during 2004–2024. A quantitative, longitudinal, and non-experimental design was applied using trade data from Trade Map, macroeconomic indicators from the World Bank, geographic distance data from CEPII GeoDist, and information on trade agreements from official sources. The analysis combined export performance indicators, normalized revealed comparative advantage, normalized relative purchasing capacity, the temporal stability of international demand, and a gravity model estimated through EGLS with PCSE correction. The results reveal a clear structural difference between the two countries. Peru increased its mango exports from USD 42.028 million in 2004 to USD 316.986 million in 2024 and maintained positive values of normalized revealed comparative advantage throughout the period. Colombia expanded its exports from USD 806 thousand to USD 15.037 million, although its competitive position became positive only from 2020 onward. The normalized relative purchasing capacity index identified the Netherlands, France, Spain, and the United States as the most important demand markets, while the stability analysis showed greater persistence among importers after 2018. The gravity model indicated that exporter GDP, destination population, and trade agreements were positively associated with exports. Overall, Peru has a consolidated export structure, whereas Colombia remains in an expansion phase and requires greater market diversification and consolidation to strengthen its international export sustainability.

## 1. Introduction

Mango occupies a prominent position among tropical fruits with a strong presence in global agri-food markets, driven by sustained demand, a broad range of cultivars, and its capacity to participate in both fresh-consumption channels and value-added agro-industrial chains. The structural limitations of this perishable product condition its internationalization capacity, because it is highly vulnerable to mechanical damage, rapid quality deterioration, difficult-to-control ripening patterns, and increasingly strict sanitary and phytosanitary protocols ( Ntsoane et al., 2019; Sivakumar et al., 2011). Within this framework, competitiveness does not refer only to exported volume, but also to maintaining traceability, food safety, logistical timeliness, and continuity of supply in highly demanding markets ( Le et al., 2022; Matulaprungsan et al., 2019).

Mango represents a strategic alternative for diversifying the agro-export supply of emerging economies such as Colombia and Peru, while also generating foreign exchange and stimulating producing territories. However, export expansion does not by itself guarantee a sustainable trajectory ( Ayyaz et al., 2019). Evidence from agri-food trade shows that export growth may occur despite structural weaknesses when countries depend on a limited number of destinations, face non-tariff barriers, or show low logistical sophistication ( Mizik, 2021; Wickrama et al., 2024). Therefore, the performance of Colombian and Peruvian mango exports should be assessed not only from the perspective of trade volume, but also through the robustness of their market structure and the stability of their competitive position ( Lădaru et al., 2024; Wang et al., 2022).

The difference between the two economies is reflected in the accumulated evidence. According to Tatiana Gonzales Cabrera et al. (2022), Peru is among the world’s leading exporters of fresh mango, and non-traditional agro-exports continue to be led by this fruit, which maintained growth despite the pandemic. Likewise, Santa María & Chacón (2023) reports that the average for 2013–2022 shows annual growth of 10% in FOB value, with the Netherlands and the United States predominating as destination markets. Even so, a certain degree of market concentration is evident. In turn, Montes Ninaquispe et al. (2024) identifies a strong export specialization indicator in Peruvian fruits, together with a problem of market and exporter diversification. In the case of Colombia, Ibañez and Colinan (2024) documented the productive growth of mango in areas such as Cesar and its effect on the local economy, although exports remain marginal compared with productive potential. Gaitan et al. (2022) warned that international competitiveness in mango requires transforming the structure of varieties, seasonality, and phytosanitary access, limitations that are not exclusive to Colombia but also affect other emerging producers. The scarcity of scientific literature on Colombian mango exports contrasts with the extensive evidence available for Peru. This, in itself, reflects the lower level of export development in the sector Ayyaz et al. (2019). The large gap between an economy with a consolidated trajectory and another in an early stage of insertion justifies an analytical comparison that evaluates the structural conditions, market diversification, relative competitiveness, and gravity-related determinants under which sustainable insertion in perishable tropical fruits can be built.

Market diversification constitutes a critical dimension for agricultural products. Concentrating exports in a small number of importers exposes value-chain actors to regulatory changes, price increases, and logistical disruptions ( Balavac & Pugh, 2016). Greater structural diversity distributes risks, reduces commercial dependence, and strengthens the resilience of the export profile. This relationship has been widely documented through the Herfindahl-Hirschman index and market-share measures Balavac and Pugh (2016), and its relevance for the analysis of fresh fruits has been confirmed in studies on destination concentration and export sustainability ( Juárez et al., 2025).

In Latin America, agro-exports have grown significantly in recent decades, while also revealing vulnerabilities associated with dependence on traditional markets and supply concentration ( Ayuda et al., 2022; Melo et al., 2014). Colombia and Peru are emerging economies with agro-export potential, but they differ in infrastructure, export experience, current trade agreements, and the degree of market consolidation. This distinction makes it relevant to compare their mango exports in order to determine which country presents a more diversified structure and a more advantageous competitive position ( Leitão, 2024; Xu et al., 2023).

The problem becomes more complex when considering that export flows do not depend solely on domestic supply. Bilateral trade is influenced by several factors, including economic size, geographic distance, transportation costs, and tariff barriers ( Leitão, 2024). In this sense, diversification analysis cannot be limited to a description of current destinations; it must also explain why exports are concentrated in certain markets and which opportunities remain underused. Gravity models are particularly useful because they connect export performance, market structure, and the economic determinants of trade ( Ayuda et al., 2022; Wang et al., 2022; Xu et al., 2023).

Therefore, the central question is not whether Colombia and Peru export mango, but whether they do so through a diversified, competitive, and economically grounded commercial trajectory. Interpreting export growth as a commercial success without considering concentration vulnerabilities or low relative competitiveness could lead to partial conclusions ( Bojnec & Fertő, 2017; Seyoum, 2007). Accordingly, it is necessary to jointly assess market share, destination concentration, net exports, revealed symmetric comparative advantage, revealed symmetric relative purchasing capacity, and gravity-related determinants of mango trade in both economies.

The literature on agri-food competitiveness has moved from approaches focused on static comparative advantages toward multidimensional perspectives that incorporate quality, productivity, innovation, and performance in international markets. Mizik (2021) notes that there is no universal measure of competitiveness in agri-food trade; therefore, it is essential to combine measures that capture specialization, trade performance, and export structure. Wang et al. (2022) and Lădaru et al. (2024) emphasize that this competitiveness also depends on technical complexity, institutional quality, and logistical infrastructure, while Suroso et al. (2023) highlights the importance of adapting to changing market requirements. This provides producers with a broader perspective for generating value from mango exports ( Jambor & Babu, 2016).

In the specific case of mango, Ayyaz et al. (2019) used revealed comparative advantage indices to compare exporting countries and found important asymmetries in competitive positions. Fruit exports are significantly affected by non-tariff barriers; according to Wickrama et al. (2024), mango export competitiveness depends on storage technologies, preventive treatments, and effective logistical coordination, as emphasized in the literature on postharvest quality ( Ntsoane et al., 2019; Sivakumar et al., 2011). Despite these advances, no studies have compared Colombia and Peru from an integrated perspective that links diversification, competitiveness, and trade gravity.

Another relevant research stream has examined export diversification as a mechanism for reducing vulnerability. Balavac & Pugh (2016) associate lower volatility in external performance with openness, diversification, and institutional quality. Barbieri et al. (2024) analyze diversification as a territorial dimension of export performance. Recent studies also indicate that the HHI can be used to evaluate market dependence and the commercial sustainability of Peruvian fresh fruits ( Juárez et al., 2025). These studies agree that diversification is not only a matter of increasing the number of destinations, but also of strengthening the export structure against external shocks.

Finally, gravity-model analysis indicates that economic size, distance, trade agreements, and access barriers affect agri-food flows. Altamirano-Gonzales and Morán-Santamaría (2026) applied this approach to Latin American agri-food exports; Melo et al. (2014) showed that sanitary standards affect fruit trade flows; and Leitão (2024) systematized the contributions of the gravity model to the analysis of international trade. This evidence supports the inclusion of this model in the analysis of Colombian and Peruvian mango exports, as it allows the identification of current market-access determinants and destinations with untapped potential ( Wang et al., 2022; Xu et al., 2023).

The theory of comparative advantage and its empirical operationalization through international trade indicators provide the basis for this study. The Balassa index indicates whether a country has positive export specialization in a given product. When compared across countries, revealed symmetric comparative advantage corrects the asymmetries of the traditional index and facilitates comparisons between countries and periods ( Laursen, 2015). In this study, the RSCA is particularly suitable for determining whether Colombia and Peru hold a favorable competitive position in global mango trade during 2004–2024 ( Ayyaz et al., 2019).

Complementarily, market share approximates each country’s relative importance in world trade; the Herfindahl-Hirschman index measures the degree of concentration or diversification of export destinations; and the net export index distinguishes clearly exporting profiles from importing profiles ( Bojnec & Fertő, 2017; Mizik, 2021). Combining these indicators with the gravity model enables a more comprehensive reading of mango exports by incorporating observed performance, market structure, relative competitiveness, and bilateral trade determinants ( Ayuda et al., 2022).

Despite the advances noted above, a research gap remains. The literature has already examined mango competitiveness, export diversification, market concentration, and the gravity determinants of agricultural trade; however, comparative evidence that integrates market share, the Herfindahl-Hirschman index, the net export index, revealed symmetric comparative advantage, normalized relative purchasing capacity, and the gravity model within the same analytical framework remains scarce for evaluating mango exports from Colombia and Peru during 2004–2024. This absence prevents a clear determination of whether both emerging economies have built a diversified and competitive international insertion or whether their performance still depends on vulnerable trade structures. Accordingly, this study aims to evaluate market diversification and the export competitiveness of mango in Colombia and Peru. Through trade indicators, it analyzes the commercial performance of these emerging economies; through trade-based models, it evaluates export concentration and revealed comparative advantage; and, finally, through a gravity model, it assesses the diversification of destination markets for exports made by these emerging economies during 2004–2024.

## 2. Methods

### 2.1 Approach, Type, Design, and Scope

The study adopted a quantitative approach because it analyzes the competitive performance of mango exports from Colombia and Peru through the objective measurement of trade variables, the estimation of standardized indicators, and the empirical verification of relationships using statistical and econometric techniques, which allows replicable and comparable results across countries and periods ( Lim, 2025). Its purpose was applied, as it was oriented toward generating useful evidence for decision-making in the agro-export sector of both countries ( Hernández et al., 2024). The design was non-experimental and longitudinal, with variables observed without manipulation during 2004–2024, allowing trends and structural changes that are not observable in cross-sectional studies to be captured ( Li et al., 2024). The scope combined a descriptive dimension, aimed at characterizing export behavior and market structure, with an explanatory dimension, focused on estimating bilateral trade determinants through a gravity model, following an appropriate approach for studies on export sustainability ( Llerena Tapia et al., 2025).

### 2.2 Unit of Analysis, Population, and Sample

The unit of analysis was international trade in mango exports from Colombia and Peru; the unit of observation corresponded to annual flows to each destination market during 2004–2024. The population comprised all export records under tariff subheading 0804502000 (fresh or dried mangoes and mangosteens) in Trade Map. Because the study used the complete set of available observations, a census sample was employed, which ensures the exact calculation of the HHI, TC, VCRN, and CRCN without selection bias and allows the gravity model to capture all active bilateral trade relationships in the period ( Shukla et al., 2023).

### 2.3 Product Delimitation and Information Sources

The study focused exclusively on fresh and dried mangoes, excluding juices, preserves, and other processed forms, whose trade dynamics, distribution channels, and international prices differ substantially ( Barmon et al., 2025). The empirical information was obtained from the sources described in Table 1.

**Table 1.** Data, variables, and sources.

Variable	Definition	Source
Exp	Mango exports from Colombia/Peru to the destination country (USD FOB).	Trade Map (ITC, 2024)
PIB_origen	GDP of the exporting country (Colombia or Peru), constant dollars.	Banco Mundial (2024)
PIB_destino	GDP of the destination importing country, constant dollars.	Banco Mundial (2024)
Pop_dest	Total population of the destination country.	Banco Mundial (2024)
Dist	Geographic distance (km) between the economic centers of the exporter and the destination.	CEPII GeoDist (Mayer & Zignago, 2011)
TLC	Dummy: 1 if an FTA is in force between the exporter and the destination; 0 otherwise.	MINCETUR/MINCIT/OMC

### 2.4 Variables and Indicators

To evaluate market diversification and the competitive performance of mango exports from Colombia and Peru, four complementary indicators were combined: market concentration (HHI), trade profile (TC), revealed comparative advantage (RCA and RSCA), and geographic potential (gravity model). This combination responds to the fact that no individual index fully captures the complexity of agri-food competitiveness ( Barmon et al., 2025).

***2.4.1 Procedures***

*Herfindahl-Hirschman Index (HHI)*

The HHI simultaneously measures the concentration and geographic diversification of export flows, as it is sensitive both to the number of destinations and to the equity of their distribution ( Hu et al., 2023). It is calculated as follows:

$${\mathrm{HHI}}_{t}=\sum s_{i,t}^{2}$$

where:

${\mathrm{HHI}}_{t}$ = market concentration index of the exporting country in year *t*.

$s_{i,t}$ = share of destination country *i* in total mango exports in year *t*, calculated as:

$$s_{i,t}=\frac{X_{i,t}}{\sum X_{i,t}}$$

$X_{i,t}$ = value of mango exports to destination *i* in year *t*.

n = total number of active destination markets in year *t*.

The index ranges from 0 to 1. For interpretation, the criteria of the U.S. Department of Justice (DOJ) are used: HHI <1,500, low concentration; 1,500 to 2,500, moderate concentration; and >2,500, highly concentrated market (Antitrust Division, 2026).

*Trade Competitiveness Index (TC)*

The TC examines the net position of Colombia and Peru in mango trade based on their exports and imports. This makes it possible to determine whether the country is a net exporter or a net importer, a dimension not captured by the RCA, and allows evidence to be triangulated from different methodological perspectives ( Long, 2021).

$${\mathrm{TC}}_{t}=\frac{X_{t}-M_{t}}{X_{t}+M_{t}}$$

where:

${\mathrm{TC}}_{t}$ = trade competitiveness index of the exporting country in year *t*.

$X_{t}$ = value of mango exports of the exporting country in year *t*.

$M_{t}$ = value of mango imports of the same country in year *t*.

The TC takes values in the interval [−1, 1]. Values close to +1 indicate a consolidated net exporter, whereas values close to −1 indicate a net importer.

Revealed Comparative Advantage Index (RCA) and Symmetric Version (RSCA).

The RCA determines whether export specialization is favorable relative to the world average ( Balassa, 1965), but it presents statistical asymmetry because it has no upper bound, which prevents comparisons between countries and periods ( Laursen, 2015). Therefore, the RSCA is adopted as the key measure, because it corrects this asymmetry by limiting the index to [−1, 1] and improves comparability.

$${\mathrm{RCA}}_{i,t}=\frac{\frac{X_{i,t}}{X_{t}}}{\frac{X_{i,t}}{X_{t}}}$$

where:

${\mathrm{RCA}}_{i,t}$ = revealed comparative advantage index for product *i* in year *t*.

$X_{i,t}^{p}$ = exports of product *i* by country *p* (Colombia or Peru) in year *t*.

$X_{t}$ = total exports of country *p* in year *t*.

$X_{i,t}^{w}$ = world exports of product *i* in year *t*.

$X_{t}$ = total world exports in year *t*.

Values greater than 1 indicate revealed comparative advantage. Subsequently, the RCA was transformed into RSCA as follows:

$${\text{RSCA}}_{i,t}=\frac{{\mathrm{RCA}}_{i,t}-1}{{\mathrm{RCA}}_{i,t}+1}$$

where:

${\text{RSCA}}_{i,t}$ = revealed symmetric comparative advantage of product *i* in year *t*.

${\mathrm{RCA}}_{i,t}$ = revealed comparative advantage index calculated in the previous equation.

The RSCA ranges from −1 to +1. Positive values indicate revealed comparative advantage, whereas negative values indicate relative disadvantage ( Török et al., 2020).

*Gravity Model of International Trade*

The gravity model identifies the structural factors that determine mango export flows from Colombia and Peru and quantifies untapped export potential in each destination, a dimension that static indicators cannot reveal (Ismaiel Ali Ismaiel et al., 2023). Its theoretical foundation derives from Anderson, Helpman, and Tinbergen, and it has been widely applied in studies of Latin American agro-exports ( Jadhav & Ghosh, 2024). The baseline specification is:

$$X_{\mathrm{ij}}=A\cdot \frac{Y_{i}\cdot Y_{j}}{D_{\mathrm{ij}}}$$

where:

$X_{\mathrm{ij}}$ = value of exports from country *i* to country *j*.

$Y_{i},Y_{j}$ = GDP of the exporting country *i* and importing country *j*, respectively.

$D_{\mathrm{ij}}$ = bilateral geographic distance between *i* and *j*, representing trade costs.

A = constant grouping factors common to all trade relationships.

α,β,γ = empirically estimated parameters reflecting trade sensitivity to changes in economic size and distance.

Transformed into its log-linearized form for econometric estimation:

Estimated log-linear specification

$$\ln\left({\mathrm{EXP}}_{\mathrm{ijt}}\right)=\beta_{0}+\beta_{1}\;\ln\left({\mathrm{PIB}}_{\mathrm{it}}\right)+\beta_{2}\;\ln\left({\mathrm{PIB}}_{\mathrm{jt}}\right)+\beta_{3}\;\ln\left({\mathrm{POP}}_{\mathrm{jt}}\right)+\beta_{4}\;\ln\left({\text{DIST}}_{\mathrm{ij}}\right)+\beta_{5}{\mathrm{TLC}}_{\mathrm{ij}}+u_{\mathrm{ij}}+\varepsilon_{\mathrm{ijt}}$$

where:

$\ln\left({\mathrm{EXP}}_{\mathrm{ijt}}\right)$ = natural logarithm of mango exports from country *i* to country *j* in year *t*.

$\ln\left({\mathrm{PIB}}_{\mathrm{it}}\right)$ = logarithm of GDP of the exporting country (Colombia or Peru) in year *t*.

$\ln\left({\mathrm{PIB}}_{\mathrm{jt}}\right)$ = logarithm of GDP of destination importing country *j* in year *t*.

$\ln\left({\mathrm{POP}}_{\mathrm{jt}}\right)$ = logarithm of the total population of destination country *j* in year *t*.

$\ln\left({\text{DIST}}_{\mathrm{ij}}\right)$ = logarithm of geographic distance in kilometers between exporting country *i* and destination *j*.

${\mathrm{TLC}}_{\mathrm{ij}}$ = dummy variable: it takes the value of 1 if an FTA is in force between *i* and *j*, and 0 otherwise.

$u_{\mathrm{ij}}$ = unobserved specific effect of bilateral pair *i-j* (fixed or random effect).

$\varepsilon_{\mathrm{ijt}}$ = idiosyncratic error term.

*Estimator Selection and Hausman Test*

Three specifications were estimated: Pooled OLS, which ignores individual heterogeneity; FE, which controls time-invariant unobserved heterogeneity; and RE estimated through EGLS under Swamy-Arora variance, which is more efficient when individual effects are not correlated with the regressors. The Hausman test ( Table 2) was applied, and its null hypothesis (p > 0.05) was not rejected. In the presence of heteroscedasticity or autocorrelation, standard errors were corrected using PCSE Period SUR ( Davidescu et al., 2022). Robustness tests were also performed by excluding 2005–2007 and by estimating specifications without the population variable; in both cases, the coefficients retained their sign and significance. Zero flows were replaced with a minimum positive constant to allow logarithmic transformation ( Ismaiel Ali Ismaiel et al., 2023). All estimations were conducted in EViews 13.

**Table 2.** Hausman Test.

Test Summary	Chi-Sq. Statistic	Chi-Sq. d.f.		Prob.
Cross-section random	45.321017	3		0
**Variable**	**Fixed**	**Random**	**Var (Diff.)**	**Prob.**
LN_GDP_ORIGEN	5.322426	6.364009	0.106846	0.0014
LN_GDP_DEST	−0.196279	0.284043	0.008701	0
LN_POP_DEST	9.020208	1.534887	3.511334	0.0001

*Market Potential Calculation*

The coefficients of the RE model were used to project potential mango exports from Colombia and Peru to each destination. To correct logarithmic retransformation bias, the smearing estimator (Zhang et al., 2025) was applied, using the mean of the exponential of the residuals as a correction factor:

$$\overset{-}{E}X{\hat{P}}_{\mathrm{ijt}}={\hat{e}}^{\ln\left({\text{EXP}}_{\text{ijt}}\right)}\times e^{u_{\mathrm{ij}}}$$

where:

$\overset{-}{E}X{\hat{P}}_{\mathrm{ijt}}$ = estimated potential exports from country *i* to destination *j* in year *t*.

${\hat{e}}^{\ln\left({\text{EXP}}_{\text{ijt}}\right)}$ = model prediction on the logarithmic scale, converted to levels using the exponential function.

$e^{u_{\mathrm{ij}}}$ = smearing correction factor, calculated as the mean of the exponential of the residuals from the random-effects model.

Market potential was calculated by comparing potential exports with observed exports:

$${\text{Potencial}}_{\mathrm{ijt}}=\frac{\overset{-}{E}X{\hat{P}}_{\mathrm{ijt}}-{\mathrm{EXP}}_{\mathrm{ijt}}}{{\mathrm{EXP}}_{\mathrm{ijt}}}\times 100$$

where:

${\text{Potencial}}_{\mathrm{ijt}}$ = gap between potential and observed exports, expressed as a percentage.

$\overset{-}{E}X{\hat{P}}_{\mathrm{ijt}}$ = potential exports estimated by the gravity model.

${\mathrm{EXP}}_{\mathrm{ijt}}$ = exports actually observed from country *i* to destination *j* in year *t*.

A positive value indicates that the country exports below its estimated potential (untapped opportunity). A value close to zero indicates trade consistent with the model fundamentals. A negative value reflects a saturated market. The market potential classification is in Table 3.

**Table 3.** Market Potential Classification.

Classification	Criterion	Interpretation
Opportunity	Potencial >50	High expansion capacity; exports far below the estimated potential.
Emerging	10 < Potencial ≤50	Opportunityes de crecimiento moderadas.
Consolidated	−10 ≤ Potencial ≤10	Balanced market; actual trade consistent with expected levels.
Saturated	Potencial < −10	Overexploited market; limited room for expansion.

*Data Processing and Statistical Analysis*

Mango export records from Colombia and Peru were organized and standardized in Microsoft Excel. The HHI and TC were calculated in IBM SPSS Statistics, a standard software package for descriptive and inferential analysis in economic and social research ( Masuadi et al., 2021). The gravity model and panel-data estimations were processed in EViews version 13, a reference tool for estimating complex econometric models with panel data in international trade studies ( Davidescu et al., 2022).

The dataset can be found in the ZENODO repository, under the title “Dataset: Market Diversification and Mango Exports in Emerging Economies: Evidence from Peru and Colombia, 2004−2024” ( Cosio et al., 2026), https://doi.org/10.5281/zenodo.20496337, License “ Creative Commons Attribution 4.0 International”.

## 3. Results

Table 4 presents the evolution of mango exports from the emerging economies of Peru and Colombia during 2004–2024. The results show a marked difference in the export scale of the two countries. Peru maintained a considerably higher export position throughout the period, increasing from USD 42.028 million in 2004 to USD 316.986 million in 2024. In terms of volume, Peruvian exports increased from 59,828 tons in 2004 to 177,670 tons in 2024, although the highest exported volume was recorded in 2021, with 253,042 tons.

**Table 4.** Mango exports from the emerging economies of Peru and Colombia to the world.

Year	Exports - Peru					Exports - Colombia				
	FOB USD - Thousand	Net Weight (MT)	Growth Rate (%)	FOB Price (USD/kg)	VCRN - Peru	FOB USD - Thousand	Net Weight (MT)	Growth Rate (%)	FOB Price (USD/kg)	VCRN - Colombia
2004	42,028	59,828		0.70	0.96	806	2,275		0.35	−0.16
2005	38,396	57,618	−9%	0.67	0.94	360	115	−55%	3.13	−0.59
2006	59,325	82,685	55%	0.72	0.95	455	126	26%	3.60	−0.55
2007	63,360	82,675	7%	0.77	0.94	626	220	38%	2.84	−0.48
2008	64,129	82,696	1%	0.78	0.94	691	374	10%	1.85	−0.54
2009	70,928	69,189	11%	1.03	0.93	340	131	−51%	2.60	−0.71
2010	90,037	97,504	27%	0.92	0.93	434	198	28%	2.19	−0.63
2011	115,414	124,051	28%	0.93	0.93	512	354	18%	1.45	−0.71
2012	117,277	99,609	2%	1.18	0.92	258	52	−50%	4.96	−0.88
2013	132,681	127,186	13%	1.04	0.93	631	210	145%	3.01	−0.80
2014	139,347	120,777	5%	1.15	0.94	684	110	8%	6.21	−0.79
2015	195,874	133,930	41%	1.46	0.96	1,094	283	60%	3.86	−0.60
2016	199,361	161,135	2%	1.24	0.95	2,207	864	102%	2.55	−0.34
2017	191,772	162,938	−4%	1.18	0.93	2,804	872	27%	3.21	−0.37
2018	246,701	199,336	29%	1.24	0.94	3,722	889	33%	4.19	−0.25
2019	252,049	191,417	2%	1.32	0.93	5,856	1,649	57%	3.55	−0.10
2020	280,542	239,391	11%	1.17	0.95	9,387	2,281	60%	4.12	0.20
2021	315,636	253,042	13%	1.25	0.94	13,785	2,396	47%	5.75	0.32
2022	296,211	249,786	−6%	1.19	0.94	13,559	2,393	−2%	5.67	0.26
2023	254,734	196,856	−14%	1.29	0.92	14,434	3,010	6%	4.80	0.29
2024	316,986	177,670	24%	1.78	0.92	15,037	3,312	4%	4.54	0.28
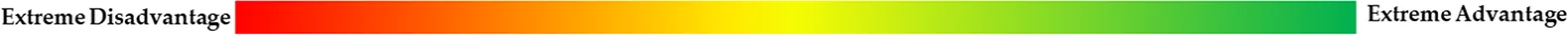										

Note. International trade data records and calculations obtained from Trade Map of the International Trade Centre (ITC).

The Peruvian series shows an overall upward trend despite contractions in specific years. The largest annual increases were recorded first in 2006, with growth of 55%, and later in 2015, with 41%. By contrast, decreases occurred in 2005, 2017, 2022, and 2023. The decline in 2023 was particularly important, as FOB value fell by 14% compared with 2022, while in 2024 it recovered by 24%. The FOB price also increased over the period, rising from USD 0.70/kg in 2004 to USD 1.78/kg in 2024, the highest value in the entire series.

Colombia followed a different export trajectory. Colombian mango exports increased from USD 806 thousand in 2004 to USD 15.037 million in 2024. Although Colombia’s figure remained far below Peru’s scale, the series shows more dynamic growth from 2015 onward. Colombian exports exceeded one million dollars in 2015 and reached USD 9.387 million in 2020, remaining above USD 13 million from 2021 onward. Exported volume also increased, from 2,275 tons in 2004 to 3,312 tons in 2024.

The Colombian FOB price was higher than the Peruvian price in most years, although this did not translate into a comparable export scale. Colombia’s price rose from USD 0.35/kg in 2004 to USD 4.54/kg in 2024, with a maximum of USD 6.21/kg in 2014. This behavior indicates that Colombia’s export growth occurred in a context of lower volumes and higher unit values, whereas Peru combined higher volumes with a more consolidated export base.

The normalized revealed comparative advantage values reported in Table 1 show a clear difference between Peru and Colombia. Peru maintained high positive VCRN values throughout the period, ranging from 0.92 to 0.96. This indicates a stable and persistent revealed comparative advantage in mango exports during 2004–2024.

Colombia followed a weaker trajectory, although with progressive improvement. Between 2004 and 2019, its VCRN values remained negative, moving from −0.16 in 2004 to −0.10 in 2019. The lowest value was recorded in 2012, at −0.88. However, from 2020 onward, Colombia reported positive VCRN values: 0.20 in 2020, 0.32 in 2021, 0.26 in 2022, 0.29 in 2023, and 0.28 in 2024.

These results indicate that Peru sustained a consolidated competitive position throughout the period, whereas Colombia moved from a position of revealed comparative disadvantage to a positive, although still moderate, position in recent years. The gap between the two countries remains considerable, especially in FOB value, exported volume, and comparative-advantage stability.

Table 5 reports the normalized relative purchasing capacity of the main international mango importers. The results show that the Netherlands maintained the highest and most stable positive values during the period, increasing from 0.74 in 2004 to 0.84 in 2024, with values above 0.80 in almost all years. This behavior positions the Dutch market as one of the most consistent demand destinations in the sample.

**Table 5.** Normalized relative purchasing capacity of international mango demand for exports from the emerging economies of Peru and Colombia (2004–2024).

N°	Importers	2004	2005	2006	2007	2008	2009	2010	2011	2012	2013	2014	2015	2016	2017	2018	2019	2020	2021	2022	2023	2024
1	United States of America	0.50	0.50	0.40	0.47	0.51	0.38	0.42	0.44	0.40	0.42	0.41	0.34	0.32	0.30	0.24	0.25	0.30	0.32	0.34	0.36	0.39
2	Netherlands	0.74	0.80	0.84	0.85	0.86	0.89	0.88	0.87	0.88	0.86	0.86	0.88	0.87	0.88	0.88	0.86	0.86	0.85	0.84	0.82	0.84
3	France	0.41	0.25	0.39	0.28	0.23	0.26	0.41	0.45	0.49	0.46	0.46	0.47	0.55	0.59	0.66	0.64	0.58	0.57	0.58	0.67	0.66
4	Spain	0.32	0.25	0.09	0.21	0.24	−0.15	0.18	0.17	0.23	0.23	0.16	0.12	0.18	0.17	0.11	0.25	0.35	0.37	0.43	0.46	0.45
5	Germany				−0.97								−1.00	−0.37	−0.44	0.17	0.41	0.31	0.46	0.44	0.38	0.36
6	United Kingdom	−0.06	0.22	0.33	0.31	0.29	0.13	0.18	0.27	0.40	0.43	0.42	0.46	0.39	0.34	0.35	0.25	0.12	0.16	0.18	0.11	0.19
7	Canada	0.31	−0.04	0.16	−0.46	−0.28	0.00	0.00	0.04	0.12	0.07	0.08	0.07	0.12	0.08	0.12	0.11	−0.07	0.04	0.02	0.12	0.05
8	Italy							−0.74	−0.63	−0.97	−0.97	−0.93	−0.94	−0.81	−0.47	0.09	0.06	0.44	0.35	0.34	0.11	0.30
9	Russian Federation	−0.98	−0.93		−0.65	−0.11	0.02	0.54	0.56	0.59	0.74	0.77	0.66	0.72	0.72	0.70	0.67	0.76	0.70	0.50	0.68	0.66
10	Polonia	−0.57	−0.89	−0.20	−0.87	−0.71	−0.69	−0.45	−0.66	−0.50	−0.70	−0.65	−0.56	−0.60	−0.68	−0.87	−0.45	−0.18	−0.12	−0.06	0.08	−0.24
11	Japan	−0.82	−0.76	−0.93	−0.94	−0.93	−0.92	−0.71	−0.62	−0.50	−0.33	−0.37	−0.43	−0.47	−0.46	−0.67	−0.69	−0.74	−0.67	−0.68	−0.64	−0.70
12	China		−1.00						−0.98				−0.83	−0.44	−0.38	−0.41	−0.40	−0.33	−0.42	−0.44	−0.28	−0.54
13	Chile	−0.99	−0.99	−0.98	−1.00	−0.98	−0.98	−0.87	−0.91	−0.76	−0.36	−0.12	−0.11	−0.04	−0.11	−0.23	−0.25	−0.60	−0.54	−0.47	−0.43	−0.39
14	Belgium										−0.90	−0.98	−0.92	−0.82	−0.89	−0.89	−0.77	−0.60	−0.37	−0.41	−0.47	−0.55
15	Switzerland				−0.93			−0.91	−0.33	−0.67	−0.54	−0.72	−0.82	−0.88	−0.80	−0.69	−0.62	−0.69	−0.58	−0.53	−0.74	−0.80
16	United Arab Emirates	−0.99	−0.99	−0.99	−0.98	−0.99	−0.97	−0.93	−0.92	−0.91	−0.92	−0.62	−0.67	−0.65	−0.49	−0.61	−0.65	−0.75	−0.77	−0.81	−0.77	−0.83
17	Norway	0.09	0.16	−0.63	−0.16	−0.18	−0.52							−0.70	−0.40	−0.14	−0.23	0.24	−0.34	0.02	−0.21	0.34
18	Denmark				−0.80	−0.61	−0.53	−0.78			−0.98					−0.87	−0.26	−0.06	0.14	0.44	0.30	0.23
19	Hong Kong, China	0.57	0.65	0.49	0.63	0.56	0.26	0.47	0.51	0.54	0.50	0.30	0.36	−0.01	0.25	0.25	0.04	0.12	−0.10	0.21	0.27	0.05
20	Argentina																−0.64	0.37	0.65	0.54	0.52	0.08
	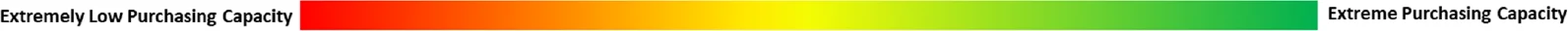																					

Note. International trade data records and calculations obtained from Trade Map of the International Trade Centre (ITC).

The United States also reported positive values throughout the period, although with a downward trend, from 0.50 in 2004 to 0.39 in 2024. France showed a more favorable trajectory, increasing from 0.41 in 2004 to 0.66 in 2024, with particularly high values since 2016. Spain also improved over time, rising from 0.32 in 2004 to 0.45 in 2024, despite the negative value recorded in 2009.

Other European markets presented heterogeneous patterns. Germany recorded missing or negative values in the early years, but reached positive values in the most recent period, closing at 0.36 in 2024. Italy also moved from negative to positive values after 2018, reaching 0.30 in 2024. Denmark showed positive values in the final years, including 0.23 in 2024.

Several markets maintained persistent negative values or weak relative demand positions. Japan remained in negative territory throughout the period and closed at −0.70 in 2024. China also maintained negative values in the available years, with −0.54 in 2024. Chile, Belgium, Switzerland, and the United Arab Emirates mainly recorded negative values, indicating lower relative purchasing capacity for mango according to this indicator.

Table 6 evaluates the temporal stability of the CRCN index using 2024 as the reference year. The results show that the relationship between historical index values and the structure observed in 2024 was positive and statistically significant in all years analyzed. Beta coefficients remained positive throughout the period, with p-values below 0.05, indicating that the relative distribution of international purchasing capacity was not random but retained a certain degree of temporal persistence. Regarding stability properties, the levels were moderate in the early years from 2004 to 2017, with R ^2^ values ranging from 0.280 to 0.493. This suggests a demand structure less aligned with that observed in 2024. By contrast, in more recent years, CRCN stability clearly strengthened. R ^2^ values increased from 0.560 in 2018 to 0.926 in 2020 and remained at 0.807, 0.894, and 0.849 in 2021, 2022, and 2023, respectively. Likewise, the R coefficient exceeded 0.875 from 2019 onward, demonstrating greater persistence in the hierarchy of importing markets. These results are consistent with the previous CRCN analysis, in the sense that markets with greater relative purchasing absorption (the United Kingdom, France, Spain, and the United States) tend to retain relevant positions over time, whereas destinations with negative or weak values are less fluid and stable as strategic markets for export expansion.

**Table 6.** Summary of the multiple linear regression model applied to Peru.

Ref. 2024	Lag	Alpha	Beta	P-Values	R ^**2**^	R	Beta/R
2004	20	20.584	0.421	0.010	0.315	0.561	0.750
2005	19	21.414	0.426	0.009	0.323	0.568	0.750
2006	18	20.944	0.403	0.017	0.280	0.529	0.762
2007	17	24.705	0.487	0.003	0.392	0.626	0.778
2008	16	24.806	0.545	<0.001	0.479	0.692	0.788
2009	15	28.853	0.592	<0.001	0.493	0.702	0.843
2010	14	21.262	0.506	0.002	0.430	0.656	0.771
2011	13	17.642	0.442	0.008	0.334	0.578	0.765
2012	12	16.810	0.422	0.008	0.327	0.572	0.738
2013	11	14.561	0.394	0.016	0.280	0.529	0.745
2014	10	14.432	0.394	0.020	0.267	0.517	0.762
2015	9	14.639	0.402	0.020	0.266	0.516	0.779
2016	8	13.513	0.576	0.003	0.408	0.639	0.901
2017	7	13.292	0.556	0.003	0.392	0.626	0.888
2018	6	11.978	0.657	<0.001	0.560	0.748	0.878
2019	5	9.140	0.900	<0.001	0.765	0.875	1.029
2020	4	0.650	0.977	<0.001	0.926	0.962	1.016
2021	3	−0.491	0.926	<0.001	0.807	0.898	1.031
2022	2	−4.742	1.012	<0.001	0.894	0.946	1.070
2023	1	−3.715	0.965	<0.001	0.849	0.921	1.048

Table 7 presents the results of the EGLS estimator with PCSE correction for 2015–2024. The coefficient of exporter-country GDP was positive and statistically significant (beta = 6.364; p = 0.000), indicating that the economic size of the exporting country was positively associated with mango export flows. The coefficient of destination-country GDP was also positive but not statistically significant (beta = 0.284; p = 0.256). Therefore, in this specification, destination GDP did not show a statistically significant relationship with mango exports. By contrast, the population of the destination country had a positive and significant coefficient (beta = 1.535; p = 0.0003), suggesting that more populous markets were associated with larger export flows.

**Table 7.** Results of the Gravity Model Estimated Using EGLS with PCSE Correction (2015–2024).

Variable	Coefficient	Std. Error	*t-Statistic*	Prob.
C	−68.5126	11.68403	−5.9	0
LN_GDP_ORIGEN	6.364009	0.48719	13.1	0
LN_GDP_DEST	0.284043	0.250018	1.14	0.2561
LN_POP_DEST	1.534887	0.423127	3.63	0.0003
LN_DIST	−1.898413	1.147936	−1.7	0.0984
TLC	4.650881	1.579074	2.95	0.0033
**Effects Specification**				
			**S.D.**	**Rho**
Cross-section random			6.37	0.4833
Idiosyncratic random			6.59	0.5167
**Weighted Statistics**				
R-squared	0.127455	Mean dependent var		−0.075595
Adjusted R-squared	0.124782	S.D. dependent var		7.136237
S.E. of regression	6.676169	Sum squared resid		72740.25
F-statistic	47.67832	Durbin-Watson stat		0.949233
Prob (F-statistic)	0			
**Unweighted Statistics**				
R-squared	0.174611	Mean dependent var		−0.343481
Sum squared resid	157223.1	Durbin-Watson stat		0.439168

Note. Obtained from the model estimation in EViews 9.0.

The distance coefficient was negative, as expected in a gravity model, although only marginally significant at the 10% level (beta = −1.898; p = 0.0984). This result indicates that geo-graphic distance could reduce mango export flows, although the statistical evidence is weaker than that observed for exporter-country GDP, destination population, and trade agreements. The free trade agreement variable showed a positive and statistically significant coefficient (beta = 4.651; p = 0.0033), indicating that the existence of a trade agreement was associated with higher mango exports.

The model was globally significant, with an F-statistic of 47.678 and p = 0.000. However, the weighted R-squared was 0.127 and the adjusted R-squared was 0.125, indicating that the model explains only a limited proportion of the observed variability in mango export flows. Therefore, the estimated coefficients should be interpreted as evidence of statistical association rather than as a complete explanation of bilateral export performance.

Table 8 classifies the markets with the highest estimated expansion potential for 2024 according to the gravity model. The countries classified as ‘Opportunity’ were Australia, Ireland, Norway, Sweden, Austria, Denmark, Venezuela, Finland, the Czech Republic, and India. These destinations showed positive gaps between estimated potential exports and observed exports.

**Table 8.** Ranking of Markets with the Highest Expansion Potential According to the Gravity Model (2024).

Country_Dest	Export_Fob	Pred_Exp	Potential	Class_Cod	Classification
Australia	0.0001	1.68E+29	1.68E+35	3	Opportunity
Irlanda	0.0001	8.58E+28	8.58E+34	3	Opportunity
Norway	0.0001	3.08E+28	3.08E+34	3	Opportunity
Suecia	0.0001	2.81E+28	2.81E+34	3	Opportunity
Austria	0.0001	2.40E+28	2.40E+34	3	Opportunity
Denmark	0.0001	1.97E+28	1.97E+34	3	Opportunity
Venezuela	0.0001	8.43E+27	8.43E+33	3	Opportunity
Finlandia	0.0001	6.36E+27	6.36E+33	3	Opportunity
República Checa	0.0001	6.22E+27	6.22E+33	3	Opportunity
India	0.0001	4.41E+27	4.41E+33	3	Opportunity

Note. Obtained from the model estimation in EViews 9.0.

Australia ranked first, followed by Ireland, Norway, Sweden, and Austria. The other opportunity-potential markets were Denmark, Venezuela, Finland, the Czech Republic, and India. In all cases, the classification indicates that observed exports were below the potential estimated by the model.

Nevertheless, the magnitude of the predicted values is extremely high; therefore, these results should be interpreted with caution. The values should be understood as exploratory signals of relative market potential, not as definitive commercial projections. Replacing zero with a minimum positive constant, applying logarithmic transformation, and retransfor-mation of predicted values may have amplified the estimated gaps. Consequently, these markets should be viewed as candidates for subsequent validation rather than as immediate export targets.

## 4. Discussion

The results obtained show a clear divergence in the structure of international mango trade in Peru and Colombia during 2004–2024. Peru’s export trajectory reflects strengthening throughout the period analyzed, as well as a systematic increase in its FOB value. In Colombia, the dynamics of these flows are different, although the VCRN shows a more favorable performance by becoming positive from 2020 onward. This confirms that the two emerging economies are not at the same level of export maturity: Peru acts as a consolidated exporter, whereas Colombia is in a stage of expansion and initial positioning.

This result is consistent with previous literature on the Peruvian agro-export sector. Studies on Peruvian mango exports highlight their continued growth, presence in international markets, and role within non-traditional agro-exports ( Morán-Santamaría et al., 2026). Likewise, the literature on Peruvian agro-exports has identified significant specialization in fruits, although with limited diversification and dependence on a few markets, problems that still persist ( García-López et al., 2025). In this sense, the results of the present study not only confirm Peru’s export strength but also show that this strength should be analyzed not only through growth in exported value, but also through the structure of destination markets.

In the Colombian case, the results show a different pattern. Although Colombia increased its export value between 2004 and 2024, its share remains low compared with Peru.

The recent improvement in the VCRN indicates an evolution in the competitive position of Colombian mango; however, this progress has not yet translated into export consolidation. The literature had already noted that Colombia has productive potential; however, it faces limitations in converting that potential into solid international insertion, mainly due to issues related to varieties, seasonality, phytosanitary access, and export structure ( Cortés-Cataño et al., 2024). According to the results obtained in this study, that interpretation is confirmed: Colombia is growing, but it has not yet reached Peru’s scale or competitive stability.

There is an important difference between the two countries in terms of FOB unit prices. Colombia has higher unit prices than Peru in several years, but this does not mean that it is more competitive or has a stronger presence in the international market. The fact that a high price does not by itself guarantee a solid export position is an important finding. In mango, as in other perishable products, competitiveness also depends on maintaining volumes, ensuring supply continuity, and complying with sanitary standards. This is consistent with recent approaches that view agri-food competitiveness as multidimensional rather than merely as a relationship between price and exported volume (Bergman et al., 2025; Collantes-Barturen & Morán-Santamaría, 2025).

The results of the normalized relative purchasing capacity indicator also show important differences among destination markets. The Netherlands appears as one of the most stable markets with the highest relative purchasing capacity, while the United States records positive values, although with a less expansive trajectory. France and Spain show gradual progress, indicating that some European markets have gained importance in international mango demand. In contrast, Japan, China, Chile, Switzerland, and the United Arab Emirates recurrently show negative or low values. This suggests that export diversification is not only about reaching more destinations, but also about building a market structure with real demand, sta-bility, and absorption capacity.

According to Sangita (2018), the literature on export diversification indicates that concentration in a few markets can increase exposure to regulatory changes, price fluctuations, and other risks. This is especially important for mango because it is a perishable product with quality requirements, transportation time constraints, and sanitary standards. Therefore, a sustainable export strategy cannot be limited to selling greater volume; it must also improve the solidity of the commercial structure. At this point, Peru faces the challenge of maintaining its leadership without depending excessively on a few markets, while Colombia needs to reach more destinations without losing consistency in quality, volume, and continuity.

The model results show that exporter-country GDP, destination-country population, and the existence of trade agreements are positively associated with mango export flows. This is consistent with gravity trade theory, which argues that bilateral flows depend on economic size, potential demand, distance, and institutional access conditions ( Altamirano Gonzales & Morán Santamaría, 2026). The positive effect of trade agreements indicates that preferential-access conditions facilitate mango insertion into international markets, although they do not replace other factors such as logistics, phytosanitary compliance, and supply capacity.

Destination-country GDP was not statistically significant in the model, indicating that the importer’s aggregate economic size does not by itself explain the dynamics of mango exports. By contrast, destination population showed a positive and significant relationship. This may be interpreted as evidence that the size of the consumer market is more relevant than the total economic size of the country when analyzing a specific food product. However, this interpretation should be treated cautiously because the model has low explanatory power and therefore does not capture all determinants of bilateral mango trade.

Geographic separation showed the expected negative sign, although with marginal significance. This is relevant because mango, as a perishable product, is subject to transportation costs, logistical times, and deterioration risks throughout the export chain. Marginal significance indicates that distance does not necessarily constitute an absolute barrier. In current agri-food markets, the effect of distance may be offset by trade agreements, logistical infrastructure, postharvest technology, and coordination capacity across the export chain. This observation is consistent with studies that highlight the importance of postharvest management and logistics for the international competitiveness of mango ( Cortez-Clavo et al., 2025; Zhang & Mohammad, 2024).

The report classifies Australia, Ireland, Norway, Sweden, Austria, and Denmark as the most recommended destinations, while Venezuela, Finland, the Czech Republic, and India appear as opportunity markets. However, the extremely high projected values should be interpreted cautiously. These results are not definitive commercial projections, but exploratory signals of relative potential. The treatment of zero flows, the use of logarithmic transformations, and the retransformation of estimated values may have widened the differences between observed and potential exports. Therefore, the markets mentioned should be considered starting points for further analysis using sanitary, logistical, tariff, commercial, and effective-demand criteria.

From the perspective of export sustainability, the results suggest that mango performance should not be measured only by FOB-value growth. A sustainable pattern implies real diversification, competitive stability, access to markets with effective demand, and lower vulnerability to external shocks.

The study has several limitations that should be considered. First, the analysis uses aggregate international trade data and does not consider differences by mango variety, quality, certifications, exporting firms, or marketing channels. Second, the subheading used groups fresh or dried mangoes and mangosteens, which may conceal within-product differences. Third, the gravity model identifies relevant associations but does not fully explain the complexity of export flows. Fourth, extreme values of market potential require methodological validation before being used as a basis for strategic decisions. Future research could include firm-level information, destination-concentration analysis, specific sanitary barriers, logistical costs, and differentiation between fresh, dried, and processed mango.

## 5. Conclusions

This study examined market diversification and mango export competitiveness in two emerging countries, Peru and Colombia, during 2004–2024. The results show that each country followed a different path within international mango trade. Peru maintains a consolidated export position: it has higher FOB values, exports larger volumes, and its normalized revealed comparative advantage (VCRN) remained positive throughout the period. Colombia, in contrast, shows a more recent and smaller-scale entry, although it has been improving progressively since 2020, when its VCRN values moved into positive territory.

Peru is the country with the greatest competitive capacity in this comparison, and this is clearly reflected in its export performance. Its foreign sales grew steadily between 2004 and 2024, with some fluctuations in certain years, and reached their highest FOB value in 2024. This trajectory supports the idea of a more mature export structure, capable of maintaining international presence and generating higher trade revenues. Colombia also grew, especially in the last decade, but its performance remains more limited than Peru’s, both in export scale and competitive stability.

Comparing the VCRN of both countries reveals a structural difference between them. Peru recorded high and positive VCRN values throughout the period, reflecting persistent export specialization in mango. Colombia, by contrast, recorded negative values for most of the series. From 2020 onward, it moved into positive territory, indicating an improvement in its export positioning, although it still does not reach Peru’s level of consolidation.

The normalized relative purchasing capacity results reveal that international mango demand is concentrated in markets with different behaviors. The Netherlands stands out as one of the most stable markets with the highest relative purchasing capacity, while the United States maintains positive values, although with less dynamism in recent years. France and Spain show a favorable evolution, indicating real opportunities within the European market. By contrast, countries such as Japan, China, Chile, Switzerland, and the United Arab Emirates report negative or weak values, showing that not all destinations offer the same level of commercial opportunity.

The gravity model identified the factors associated with mango export flows. Exporter-country GDP, destination-country population, and the existence of trade agreements showed a positive and significant relationship with exports. Distance had the expected negative sign, although with marginal significance. Importer-country GDP, in contrast, was not statistically significant. These results indicate that mango export growth does not depend only on the economic size of markets, but also on the exporting capacity of the country of origin, the population size of the destination, and institutional conditions for market access.

For 2024, the ranking of expansion potential identified Australia, Ireland, Norway, Sweden, Austria, Denmark, Venezuela, Finland, the Czech Republic, and India as opportunity markets. However, the extremely high magnitude of the estimated values requires cautious interpretation. These are not definitive export projections, but exploratory signals of relative potential that require additional validation through logistical, sanitary, tariff, commercial, and effective-demand analysis.

Overall, the results allow us to conclude that Peru has built a stronger and more competitive international insertion in mango trade, whereas Colombia is undergoing a growth stage with recent progress but still faces structural challenges. For Peru, the main challenge is to maintain its competitiveness and improve the effective diversification of its markets to reduce vulnerabilities to external shocks. For Colombia, the challenge is to convert its recent improvement into a more stable export strategy, with greater scale, supply continuity, and sustained access to international markets.

Finally, the study indicates that mango export sustainability cannot be measured solely by FOB-value growth. Sustainable international insertion requires combining competitiveness, destination diversification, stability of comparative advantage, real purchasing capacity in destination markets, and favorable market-access conditions. Future research should deepen the analysis by mango varieties, exporting firms, certifications, phytosanitary barriers, logistical costs, and differences among fresh, dried, and processed mango in order to explain more precisely the conditions that determine the product’s international competitiveness.

## Data Availability

ZENODO repository. Dataset: Market Diversification and Mango Exports in Emerging Economies: Evidence from Peru and Colombia, 2004-2024. https://doi.org/10.5281/zenodo.20496337 ( Cosio et al., 2026). This project contains the following underlying data:
• DATASET. (It contains a database of the target markets from 2004 to 2024). • RESULTS. (It contains the statistical values of the results). • TABLAS_GRAVITY_MODEL. (It contains the statistical values of the application of the gravity model). Data is available under the terms of the license “ Creative Commons Attribution 4.0 International”.
