# Supplementary material for: Market Diversification and Mango Exports in Emerging Economies: Evidence from Peru and Colombia, 2004-2024
Source: F1000Res. 2026 Jun 23;15:363. Originally published 2026 Mar 5. [Version 2] doi: 10.12688/f1000research.177965.2 (PMC13555064; doi:10.12688/f1000research.177965.2)
Supplement: Peer review history

# Reviewer report: Reviewer response for version 2

**DOI:** 10.5256/f1000research.203055.r497406

**Published:** 2026-10-12

**Authors:** Lindon Vela Meléndez

**Affiliations:** 1 Universidad Nacional Pedro Ruiz Gallo, Lambayeque, Lambayeque, Peru

Version 2 of the manuscript is coherent and deserves to be indexed.

 It presents an up-to-date and relevant theoretical framework; statistical methods are robust; and provides access to data for replication, should readers wish to do so.

 The analysis of the results is exhaustive and the contributions to the current state of knowledge are evident.

 The article stands out for its relevance to understanding agricultural export competitiveness through a longitudinal comparison between Peru and Colombia from 2004 to 2024. Its main strength lies in linking export value, volume, unit prices, and comparative advantages, thereby avoiding the reduction of trade performance to mere revenue growth. The use of Trade Map, the World Bank, and CEPII, along with the declared availability of data on Zenodo, promotes transparency and facilitates future verification.

 The results document divergent trajectories: Peru maintains a persistent export specialization, while Colombia has recorded positive comparative advantages since 2020. Likewise, the analysis of importers highlights the importance of the Netherlands and the favorable performance of France and Spain. These findings provide a useful comparative framework for formulating new research questions.

 I recommend further analysis using data disaggregated by variety, company, certification, and producing region; incorporating logistics costs, phytosanitary barriers, and climate-related disruptions; and evaluating the stability of trade destinations. It is also necessary to validate the estimates of export potential before using them as trade recommendations.

Is the work clearly and accurately presented and does it cite the current literature?

Yes

If applicable, is the statistical analysis and its interpretation appropriate?

Yes

Are all the source data underlying the results available to ensure full reproducibility?

Yes

Is the study design appropriate and is the work technically sound?

Yes

Are the conclusions drawn adequately supported by the results?

Yes

Are sufficient details of methods and analysis provided to allow replication by others?

Yes

Reviewer Expertise:

economy  and education

I confirm that I have read this submission and believe that I have an appropriate level of expertise to confirm that it is of an acceptable scientific standard.

# Reviewer report: Reviewer response for version 2

**DOI:** 10.5256/f1000research.203055.r497398

**Published:** 2026-07-14

**Authors:** Somaiya Rasheed

**Affiliations:** 1 College of Economics and Management, Northwest A&F University, Yangling, Shaanxi, China

**Summary**

 The paper compares mango export performance, market diversification, and competitiveness between Peru and Colombia over 2004–2024 using trade data, several trade indicators, and a gravity model. The authors frame the contribution as an integrated assessment combining revealed comparative advantage, relative purchasing capacity, concentration indices, and gravity determinants, applied to two emerging economies at different stages of export maturity. The study finds Peru has a consolidated and persistently competitive export structure, while Colombia shows recent improvement but remains in an expansion phase with less diversification and lower scale.

**Comments**

- The Herfindahl-Hirschman Index and the Trade Competitiveness Index are described in the methodology as primary instruments for assessing market concentration and net trade position, yet no HHI or TC values appear anywhere in the results. This is an omission, because the paper’s title and stated aim center on diversification, and the reader cannot evaluate that claim without the promised concentration measures.
- The variable labelled VCRN in the results (Table 4 text) is never defined in the paper. The methodology section introduces RCA and its symmetric version RSCA, but VCRN is not mentioned.
- The gravity model (Table 7) yields a weighted R-squared of 0.127, indicating that the model explains only about 13% of the variation in bilateral mango export flows. This low fit is not adequately discussed.
- The results section refers to “Table 1” when presenting VCRN values, but the first table in the results is labelled Table 4. The cross-reference is incorrect and confusing.
- The acronyms VCRN and CRCN appear only in the results and discussion, never in the methodology or list of variables. A consistent notation should be introduced from the start.
- Table 7 reports the gravity model for 2015–2024, but the overall period of study is 2004–2024. The rationale for restricting the estimation sample to the later sub-period is not explained.

**Recommendation**

**Minor Revision**

Is the work clearly and accurately presented and does it cite the current literature?

Yes

If applicable, is the statistical analysis and its interpretation appropriate?

Partly

Are all the source data underlying the results available to ensure full reproducibility?

Yes

Is the study design appropriate and is the work technically sound?

Yes

Are the conclusions drawn adequately supported by the results?

Yes

Are sufficient details of methods and analysis provided to allow replication by others?

Yes

Reviewer Expertise:

Agriculture economics, Resource and environmental economics, Behavioural and Development economics

I confirm that I have read this submission and believe that I have an appropriate level of expertise to confirm that it is of an acceptable scientific standard, however I have significant reservations, as outlined above.

# Reviewer report: Reviewer response for version 2

**DOI:** 10.5256/f1000research.203055.r497403

**Published:** 2026-07-13

**Authors:** Antony Cristhian Gonzales-Alvarado

**Affiliations:** 1 Universidade Federal do ABC, Santo André, State of São Paulo, Brazil

The manuscript entitled **“Market Diversification and Mango Exports in Emerging Economies: Evidence from Peru and Colombia, 2004–2024”** presents, in general terms, an adequate structure and addresses a relevant topic within the context of international agricultural trade. I consider that the study has potential for indexing; however, it presents some minor observations that should be addressed before final approval.

 First, since the manuscript refers to mango as a commercially important tropical fruit, I recommend incorporating its scientific name ( *Mangifera indica* L.) and expanding the description of its main botanical, agronomic, and commercial characteristics. Likewise, it would be appropriate to include a specific section in the Introduction describing the attributes that make mango a product with high export potential, considering aspects such as organoleptic quality, acceptance in international markets, nutritional value, varietal diversity, and global demand.

 In addition, this section should be complemented with a general description of the main production regions in Peru and Colombia, highlighting their agroclimatic conditions and productive characteristics that favor mango production for export markets.

 Regarding the presentation of the results, Table 2 shows probability values reported as 0. It is recommended to express these values as **<0.01**, following international statistical reporting conventions and avoiding the interpretation of a probability value as exactly zero. Likewise, Tables 4 and 5 contain some cells without reported values; it is suggested to use the symbol “–” in cases where no value is applicable or available, in order to maintain a consistent presentation and avoid empty spaces.

 Finally, in the Conclusion section, I recommend further synthesizing the content by focusing primarily on the most relevant findings obtained in the study and including future recommendations. After addressing these observations, I consider that the manuscript will be significantly strengthened in terms of scientific quality and will be able to proceed with the approval process.

Is the work clearly and accurately presented and does it cite the current literature?

Yes

If applicable, is the statistical analysis and its interpretation appropriate?

Yes

Are all the source data underlying the results available to ensure full reproducibility?

Yes

Is the study design appropriate and is the work technically sound?

Yes

Are the conclusions drawn adequately supported by the results?

Yes

Are sufficient details of methods and analysis provided to allow replication by others?

Yes

Reviewer Expertise:

Biostatistics, Applied Statistics, Multivariate Data Analysis, Econometrics, Agricultural Economics, Environmental Economics, Scientific Research Methodology, and Bibliometric Analysis.

I confirm that I have read this submission and believe that I have an appropriate level of expertise to confirm that it is of an acceptable scientific standard.

# Reviewer report: Reviewer response for version 2

**DOI:** 10.5256/f1000research.203055.r496057

**Published:** 2026-06-27

**Authors:** Jose Carlos Montes Ninaquispe

**Affiliations:** 1 Universidad de San Martín de Porres, Chiclayo, Peru

The manuscript addresses a relevant and timely topic for international trade and agro-export competitiveness, particularly by comparing mango exports from Peru and Colombia during the 2004–2024 period. The study has potential, as it integrates market diversification, competitiveness indicators, relative purchasing capacity, demand stability, and a gravity model.

Is the work clearly and accurately presented and does it cite the current literature?

Partly

If applicable, is the statistical analysis and its interpretation appropriate?

No

Are all the source data underlying the results available to ensure full reproducibility?

Partly

Is the study design appropriate and is the work technically sound?

No

Are the conclusions drawn adequately supported by the results?

No

Are sufficient details of methods and analysis provided to allow replication by others?

No

Reviewer Expertise:

My areas of expertise include international economics, international trade, export performance, market diversification, competitiveness, applied quantitative methods, statistics, econometrics, regression analysis, and time-series analysis. I also have experience in the methodological evaluation of scientific articles, particularly in assessing statistical consistency, the validity of empirical models, and the interpretation of results in economic and business studies.

I confirm that I have read this submission and believe that I have an appropriate level of expertise to confirm that it is of an acceptable scientific standard.

# Reviewer report: Reviewer response for version 1

**DOI:** 10.5256/f1000research.196290.r467169

**Published:** 2026-04-09

**Authors:** A.Malek Hammami

**Affiliations:** 1 Food and Resource Economics Department, University of Florida Tropical Research and Education Center (Ringgold ID: 53709), Homestead, Florida, USA

**Referee Report**

**Summary of the article**

 This manuscript analyzes the relationship between market diversification, prices, and mango export performance in Peru and Colombia over the period 2004–2024. Using Trade Map data and a quantitative, correlational approach implemented in SPSS, the authors conclude that market diversification is the main determinant of export performance, while price effects are less consistent and only significant in the case of Peru.

 The topic is relevant and policy-oriented, particularly for emerging economies seeking to improve export competitiveness and resilience. The use of publicly available data and the effort to address diversification are commendable. However, several important concerns related to **methodological rigor, statistical validity, and clarity of interpretation** might limit the scientific soundness of the study in its current form.

**comments**

**1. “impact” vs. correlational design**

 The manuscript repeatedly refers to the **“impact”** of diversification and prices on exports. However, the study is explicitly defined as:

 “non-experimental, correlational” (Methods section)

 This creates a fundamental inconsistency. A correlational design does not allow causal inference.

**Required revision:**

- Replace “impact” with **“association” or “relationship”** throughout the manuscript
  OR
- Substantially strengthen the empirical strategy to support causal claims

**2. Adhoc econometric framework and risk of spurious results**

 The empirical analysis lacks sufficient detail and rigor:

- AdHOC model equations are provided (not derived from a theoretical framework)
- No discussion of functional form (e.g., levels vs. logs)
- No control variables are included (e.g., exchange rates, production shocks, policy changes)
- Time-series properties (trends, non-stationarity) are not addressed

Given that the variables (exports, prices, number of markets) are likely trending over time, the very high R² values reported (≈90%) may reflect **spurious correlations rather than meaningful economic relationships**.

**Required revision:**

- Clearly specify the empirical model
- Address time-series properties (e.g., trends, stationarity)
- Include control variables or at least discuss omitted variable bias
- Provide a more cautious interpretation of results

**3. Overinterpretation of results**

 The conclusions state that diversification is the “key factor” and that price has a significant or insignificant “impact.” However, given the limitations above, these conclusions are **not sufficiently supported by the analysis**.

**Required revision:**

- Temper conclusions
- Align interpretation with the correlational nature of the analysis
- Explicitly acknowledge limitations

**4. Theoretical framework lacks integration**

 The manuscript presents several theories (Ansoff, Krugman, Heckscher–Ohlin, Porter), but they are largely descriptive and not clearly linked to:

- Hypotheses
- Model specification
- Empirical findings

**Required revision:**

- Streamline the theoretical section
- Explicitly connect theory → hypothesis → empirical test

**5. Measurement of key variables**

 The proxy for market diversification is the **number of export destinations**, which is a very simplified measure and does not capture concentration.

**Required revision:**

- Justify the choice of this measure
- Ideally, complement it with a concentration index (e.g., HHI or entropy index)
- Clarify interpretation of FOB prices (which may reflect quality differences)

**7. Writing and clarity**

 The manuscript would benefit from significant language editing. Examples include:

- “has undergone a very changing dynamic”
- “constant research of marketing strategies”
- incorrect punctuation such as “However;”

**Recommendation:** professional language editing is strongly advised.

**7.1. Structure and repetition**

- The introduction is overly long and repetitive
- Objectives and hypotheses are restated multiple times

**Recommendation:** streamline for clarity and conciseness.

**8. Scope clarification**

- The manuscript should better justify:
  - Why Peru and Colombia were selected
  - Why mango was chosen relative to other crops

**Recommendation:** Major revisions required.

Is the work clearly and accurately presented and does it cite the current literature?

No

If applicable, is the statistical analysis and its interpretation appropriate?

No

Are all the source data underlying the results available to ensure full reproducibility?

Yes

Is the study design appropriate and is the work technically sound?

No

Are the conclusions drawn adequately supported by the results?

No

Are sufficient details of methods and analysis provided to allow replication by others?

Partly

Reviewer Expertise:

Agribusiness; Agricultural Trade; Production Economics

I confirm that I have read this submission and believe that I have an appropriate level of expertise to state that I do not consider it to be of an acceptable scientific standard, for reasons outlined above.

# Reviewer report: Reviewer response for version 1

**DOI:** 10.5256/f1000research.196290.r467170

**Published:** 2026-03-26

**Authors:** Jose Carlos Montes Ninaquispe

**Affiliations:** 1 Universidad de San Martín de Porres, Chiclayo, Peru

 From a statistical and methodological standpoint, I do not consider this manuscript suitable for indexing in its present version. The central problem is that the paper presents itself as an analysis of the “impact” of market diversification and prices on mango exports, yet the empirical strategy is not capable of identifying impact in any defensible statistical sense. The study is explicitly described as quantitative, non-experimental, correlational, and longitudinal, using annual data from 2004–2024 and relying on Pearson correlations and linear regression in SPSS. That design is insufficient for causal inference, particularly when the data are clearly time series.

 The most serious flaw is the misuse of time-series data as if the observations were independent cross-sectional cases. The manuscript analyzes annual series for destination markets, FOB price, export volume, and FOB value over 21 years, yet it reports simple Pearson correlations and OLS regressions without any evidence of stationarity testing, autocorrelation diagnostics, cointegration analysis, structural-break testing, or time-series specification. This is a fundamental error. When variables display common upward trends over time, very high correlations such as 0.756, 0.925, 0.937, 0.951, and 0.957 can easily be spurious correlations driven by trend, not substantive economic relationships. In such a setting, the reported p-values and R-squared values are not persuasive.

 This problem becomes even more evident because the figures and narrative repeatedly describe sustained growth trends in the number of destination markets, export volume, and FOB value in both countries. Peru rises from 14 to 40 markets, and Colombia from 15 to 32 markets; export values also rise markedly over the study period. Under these conditions, strong contemporaneous correlations are expected even in the absence of a meaningful structural relationship. The authors never demonstrate that the residuals are stationary, that the regressions are not spurious, or that the estimated coefficients are not simply capturing shared temporal drift.

 A second major weakness is the systematic confusion between association and causation. The manuscript repeatedly uses the language of “impact,” “influence,” and “predictor,” and even concludes that diversification “brought tangible and quantifiable benefits” to export performance. That is not justified by the design. A correlational, non-experimental model using annual aggregate data cannot establish direction of causality. It is at least equally plausible that higher export success enables entry into more markets, rather than market expansion causing export success. In other words, reverse causality and simultaneity are obvious and unaddressed.

 There is also a serious endogeneity and identity problem in the specification. The dependent variable is FOB value, while one key regressor is FOB price, and the paper itself explicitly states that FOB value is logically linked to price and exported quantity. The manuscript even acknowledges that the increase in FOB value from higher FOB price is “direct” because total value is composed of quantity multiplied by unit price. This means the regression partly re-discovers a mechanical accounting relationship, not an independent explanatory effect. As a result, the coefficient on FOB price is not a clean substantive finding but is contaminated by the construction of the dependent variable itself.

 The operationalization of market diversification is also statistically weak. The paper defines diversification simply as the number of destination markets. That is not an adequate measure of diversification in trade analysis. A country can export to many destinations and still be highly concentrated if most exports go to one or two markets. The manuscript even cites prior work using more appropriate indicators such as the Herfindahl-Hirschman Index (HHI) and RCA, yet it does not apply any concentration index itself. Therefore, the paper’s core explanatory variable is a crude count, not a valid measure of diversification intensity or dispersion.

 The paper is additionally weakened by small-sample overconfidence. All analyses are based on N = 21 annual observations. With such a small time-series sample, the effective information is even lower once serial dependence is considered. Yet the authors present extremely high R, R-squared, and adjusted R-squared values as if these were proof of robustness. In reality, with trending macro-style annual data, very high R-squared values are often a warning sign of model misspecification rather than evidence of explanatory power. The manuscript does nothing to reassure the reader that these are not inflated by common time trends.

 Equally problematic is the complete absence of basic regression diagnostics. The manuscript does not report any test or discussion of residual autocorrelation, heteroskedasticity, normality, linearity, influential observations, outliers, leverage, specification error, or model stability. No Durbin-Watson statistic is provided. No residual plots are discussed. No transformation of variables is attempted, despite the obvious scale differences and strong trending patterns in value and volume. Without these diagnostics, the regression results cannot be considered statistically credible.

 The inferential framework is also poorly justified. The authors state that the population is the universe of extracted data and that the sample is based on the period 2004–2024. In other words, they are effectively using the full observed period they defined, not a sample drawn from a broader population in any meaningful sampling sense. Yet they rely on conventional significance testing without clearly articulating the stochastic process that would justify those inferential claims. This is conceptually weak and statistically underdeveloped.

 There are also internal inconsistencies that directly undermine confidence in the results section. The paper states that the Colombian price variable in the excluded-variables analysis had beta = 0.131, t = 1.683, p = 0.110, which would justify exclusion from the final model. However, Table 9 reports beta = 0.301, t = 2.893, p = 0.010, partial correlation = 0.563, tolerance = 0.429. Those values are not consistent with the text, and in fact they mirror the Peru coefficient pattern reported earlier. This strongly suggests either a copy-paste error, a reporting error, or loss of control over the analysis. For a quantitative paper, that is a major credibility failure.

 The reporting around coefficients is also careless to the point of implausibility. For Peru, the paper states that each additional market increases FOB value by 7,269.197 thousand dollars, while each extra dollar per kilogram increases FOB value by 104,375,426 thousand dollars. Even if one allows for unit-scaling complications, the manuscript does not explain these magnitudes clearly or consistently. The interpretation is presented as straightforward substantive evidence, but the scale handling is opaque and potentially misleading. This indicates weak statistical reporting discipline.

 There are further signs of inadequate quality control. The manuscript describes the study as covering “20 years,” yet the period 2004–2024 inclusive corresponds to 21 annual observations, which is also what appears in the correlation tables. That inconsistency is basic but telling. Likewise, Figure 8, which is supposed to show Colombian FOB value, is captioned as if it referred to the item exported from Peru. These errors are not minor cosmetic issues; in a quantitative manuscript, they signal insufficient rigor in data handling and reporting.

 Substantively, the model also suffers from severe omitted-variable bias. Over a 21-year period, mango export value is likely affected by exchange rates, production shocks, weather events, phytosanitary access, freight costs, trade agreements, external demand, and domestic supply constraints. The paper itself mentions COVID-19, El Niño, U.S. market access, and logistics-related issues in the descriptive discussion, yet none of these factors are modeled. At that point, attributing most variation in FOB value to diversification and price alone is not statistically convincing.

 Finally, the manuscript overstates its comparative contribution. The title refers to “emerging economies,” but the analysis is simply two separate country-specific regressions. There is no panel model, no formal cross-country comparison of coefficients, no test of whether Peru and Colombia statistically differ, and no integrated comparative framework. The article promises a broader analytical contribution than the empirical design actually delivers.

 For all of these reasons, my formal judgment is that the manuscript should not be accepted for indexing in its current form. The problem is not a matter of minor revision. The statistical strategy is fundamentally inadequate for the claims being made. To become indexable, the study would require a substantial redesign: a valid measure of diversification, proper time-series treatment, explicit diagnostic testing, correction of internal reporting inconsistencies, more defensible model specification, and a clear abandonment of causal language unless a much stronger identification strategy is implemented.

Is the work clearly and accurately presented and does it cite the current literature?

Partly

If applicable, is the statistical analysis and its interpretation appropriate?

No

Are all the source data underlying the results available to ensure full reproducibility?

Partly

Is the study design appropriate and is the work technically sound?

No

Are the conclusions drawn adequately supported by the results?

No

Are sufficient details of methods and analysis provided to allow replication by others?

No

Reviewer Expertise:

My areas of expertise include international economics, international trade, export performance, market diversification, competitiveness, applied quantitative methods, statistics, econometrics, regression analysis, and time-series analysis. I also have experience in the methodological evaluation of scientific articles, particularly in assessing statistical consistency, the validity of empirical models, and the interpretation of results in economic and business studies.

I confirm that I have read this submission and believe that I have an appropriate level of expertise to state that I do not consider it to be of an acceptable scientific standard, for reasons outlined above.
